# Neuron–astrocyte metabolic coupling facilitates spinal plasticity and maintenance of inflammatory pain

**DOI:** 10.1038/s42255-024-01001-2

**Published:** 2024-03-05

**Authors:** Sebastián Marty-Lombardi, Shiying Lu, Wojciech Ambroziak, Katrin Schrenk-Siemens, Jialin Wang, Anna A. DePaoli-Roach, Anna M. Hagenston, Hagen Wende, Anke Tappe-Theodor, Manuela Simonetti, Hilmar Bading, Jürgen G. Okun, Rohini Kuner, Thomas Fleming, Jan Siemens

**Affiliations:** 1 Institute of Pharmacology, Heidelberg University, Heidelberg, Germany; 2 Department of Biochemistry and Molecular Biology, Indiana University School of Medicine, Indianapolis, IN USA; 3 Department of Neurobiology, Interdisciplinary Center for Neurosciences (IZN), Heidelberg University, Heidelberg, Germany; 4 Dietmar-Hopp-Metabolic Center, Division of Neuropaediatrics and Metabolic Medicine, Heidelberg University, Heidelberg, Germany; 5 Molecular Medicine Partnership Unit (MMPU), European Molecular Biology Laboratory (EMBL), Heidelberg, Germany; 6 Department of Endocrinology, Diabetology, Metabolism and Clinical Chemistry (Internal Medicine 1), Heidelberg University Hospital, Heidelberg, Germany; 7 German Center of Diabetes Research (DZD), Neuherberg, Germany; 8 Present Address: Oliver Wyman GmbH, Munich, Germany; 9 Present Address: Department of Translational Disease Understanding, Grünenthal GmbH, Aachen, Germany; 10 Present Address: Taconic Biosciences, Leverkusen, Germany

**Keywords:** Sensory processing, Metabolism, Neuroscience, Neurology

## Abstract

Long-lasting pain stimuli can trigger maladaptive changes in the spinal cord, reminiscent of plasticity associated with memory formation. Metabolic coupling between astrocytes and neurons has been implicated in neuronal plasticity and memory formation in the central nervous system, but neither its involvement in pathological pain nor in spinal plasticity has been tested. Here we report a form of neuroglia signalling involving spinal astrocytic glycogen dynamics triggered by persistent noxious stimulation via upregulation of the Protein Targeting to Glycogen (PTG) in spinal astrocytes. PTG drove glycogen build-up in astrocytes, and blunting glycogen accumulation and turnover by *Ptg* gene deletion reduced pain-related behaviours and promoted faster recovery by shortening pain maintenance in mice. Furthermore, mechanistic analyses revealed that glycogen dynamics is a critically required process for maintenance of pain by facilitating neuronal plasticity in spinal lamina 1 neurons. In summary, our study describes a previously unappreciated mechanism of astrocyte–neuron metabolic communication through glycogen breakdown in the spinal cord that fuels spinal neuron hyperexcitability.

## Abstract (web summary)

Astrocytic glycogen dynamics is necessary to support neuronal transmission of long-term inflammatory pain signals in the spinal cord.

## Main

Sensory stimuli, particularly those that are strong, long-lasting or repetitive, can trigger neuronal plasticity. These activity-dependent processes require metabolic energy to fuel ensuing structural and functional changes^1^. The resulting neurophysiological changes, which often involve alterations of synaptic properties and changing of synaptic strength, can be beneficial and allow, for example, adaptation to a changing environment. But persistent stimulation in a pathological context can also give rise to chronic pain, a maladaptive form of neuronal plasticity detrimental to health^2^.

The spinal cord is the first relay station of noxious signals, where primary afferent sensory neurons connect to projection neurons residing in the dorsal spinal horn that carry signals of potentially harmful and damaging (thus painful) stimuli to higher brain centres. A multitude of additional spinal excitatory and inhibitory neurons modulate and ‘gate’ painful signals^3^. Plastic changes within this extensive neuronal network have long been associated with pathological forms of pain^4^. In parallel, many studies have emphasized the role of non-neuronal cells, such as astrocytes and microglia, in spinal nociceptive signal processing and pain chronification. Astrocytes provide metabolic support to neurons, regulate extracellular ion composition and modulate synaptic transmission, and are thus important for physiological (homeostatic) neuronal processing in the central nervous system (CNS)^5^. Astrocytes have also been implicated in modulating nociceptive signalling along the spinothalamic axis and, in this context, they play a preeminent role in the dorsal spinal cord. In pathological contexts, spinal astrocytes have been found not only to modulate the induction of long-lasting pain but also appear to be particularly relevant for the maintenance of chronic pain states^6–9^. How spinal astrocytes and neurons interact to drive pathological pain states and whether this interaction can be therapeutically harnessed for pain therapy are important medical questions.

Energy-carrying metabolites such as lactate can modulate nociceptive signal processing in spinal circuits^10^. However, the relevance and potential source of such metabolites in pathological nociceptive processing have remained largely unknown. Moreover, genetic evidence has been missing that directly links the regulation of energy metabolism and spinal nociceptive signal processing in vivo.

Here we identify and characterize a metabolic mechanism at the interface between the peripheral nervous system and the CNS that regulates excitability of spinal neurons processing and propagating noxious information. We find that spinal astrocytes and neurons metabolically interact and that strong, long-lasting pain stimuli have pronounced effects on dynamic glycogen metabolism in astrocytes. Robust and protracted glycogen build-up in spinal astrocytes is mediated by noxious stimulation-induced transcriptional activation of Protein Targeting to Glycogen (PTG). Genetically perturbing glycogen accumulation and dynamics by deleting the *Ptg* gene from astrocytes does not affect short-term/acute processing of noxious, pain-inducing signals in mice but allows animals to recover faster from long-lasting inflammatory pain. Mechanistically, we find that spinal neuronal plasticity, induced by long-lasting inflammatory pain stimulation, requires astrocytic glycogen and energy metabolism. Our study demonstrates that astrocytic energy fuels the maintenance of long-lasting inflammatory pain states and that pharmacologic interference with spinal astrocyte–neuron energy coupling may constitute a therapeutic avenue to accelerate recovery from pathological forms of pain by inhibiting maladaptive neuronal plasticity.

## Results

### Noxious stimulation induces PTG in spinal astrocytes

**Extended Data Fig. 1:**
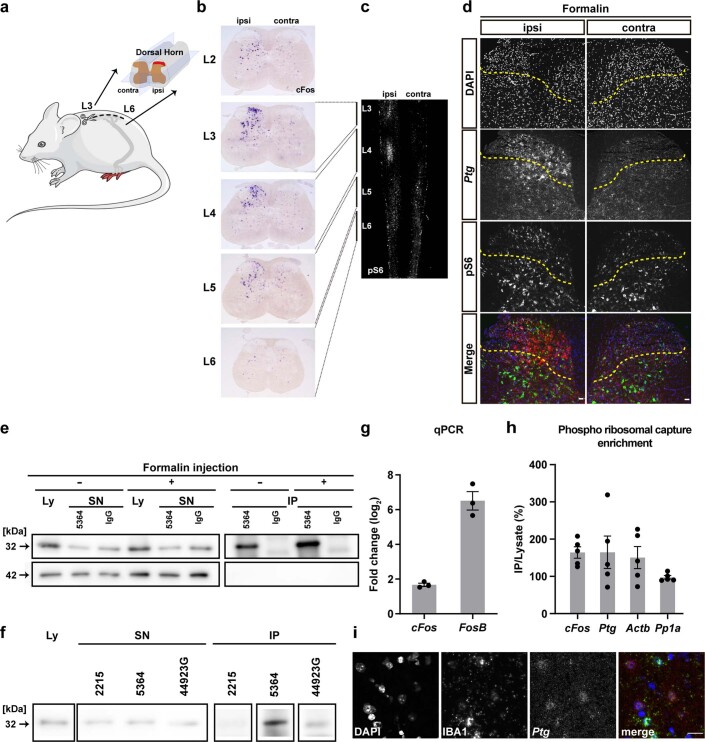
Discovery of *Ptg* as a pain-induced gene in the dorsal spinal cord (related to Fig. 1). **(a)** Cartoon illustrating the anatomy of the spinal cord and the tissue isolation of the ipsilateral (pain-affected) dorsal horn (red labeled) and the contralateral (unaffected control) dorsal horn of lumbar sections L3 to L6 for phospho-ribosome capture experiments after formalin injection into the left hindpaw. The illustration was partly generated using Servier Medical Art, provided by Servier, licensed under a Creative Commons Attribution 3.0 unported license (https://creativecommons.org/licenses/by/3.0/). **(b)** c-Fos mRNA expression (*in situ* hybridization) in the dorsal spinal cord 2 hours after intraplantar formalin injection. *C-Fos* expression is specifically detectable on the ipsilateral side (side of injection) of lumbar sections L2 to L6. **(c)** Spatial distribution of pS6 (whole mount immunofluorescence) along the spinal cord, 2 hours after intraplantar formalin injection. Maximal projection image of z-stack images of the dorsal horn shown. **(d)** Representative images of combined immunohistochemistry and fluorescent *in situ* hybridization with an antibody directed against pS6 (green) and an *in situ* probe directed against *Ptg* (red), DAPI (blue); scale bar: 40μm. **(e)** pS6 immunoprecipitation of ipsilateral (+) and contralateral (-) dorsal spinal cord 2 hours after formalin injection on the ipsilateral side. pS6 was detected in both ipsi- and contralateral lysates (Ly), reduced in supernatant (SN) and enriched in immunoprecipitation eluates (IP). IgG control did not bind any pS6. IP showed an enrichment for pS6 on the ipsilateral (+) over the contralateral (-) side. **(f)** Western blot showing anti-pS6 antibody test (Cell Signaling polyclonal antibody #2215, Cell Signaling monoclonal antibody #5364 and Invitrogen polyclonal antibody #44923 G) for pS6 immunoprecipitation using spinal cord tissue after formalin injection. Ly, the three SNs after antibody incubation and the respective IPs were loaded on one western blot gel. All three tested antibodies reduced pS6 amount in the SN after immunoprecipitation while #5364 showed best enrichment in the IP. **(g)** qPCR verification of cDNA libraries generated from phospho-ribosome captured ipsilateral and contralateral dorsal horn spinal cord tissue after formalin stimulation, amplifying *cFos* and *FosB* cDNAs. *cFos* and *FosB* showed significant and consistent enrichment in tissue isolated from the ipsilateral (formalin affected) side (N = 3 biologically independent samples derived of 3 mice). Data represent mean ± s.e.m. **(h)** Comparison of phospho-ribosomal capture to mRNA extracted from lysate. qPCR was performed before and after phospho-ribosomal capture using ipsilateral and contralateral dorsal spinal cord tissue of mice injected with formalin on the ipsilateral side. Note that the indicated genes (except for *Pp1a*) were found to be enriched (showed a higher IP/Ly ratio) in the phosphoribosomal capture (IP) condition compared to qPCRs done from ipsilateral tissue (Ly), demonstrating that phospho-ribosomal capture is more sensitive, albeit only slightly, in pulling out induced mRNA transcripts. Data represent mean ± s.e.m. **(i)** Representative images of combined immunohistochemistry and fluorescent *in situ* hybridization with an antibody directed against microglia marker IBA1 (green) and an *in situ* probe directed against *Ptg* (red), DAPI (blue); scale bar: 20μm.

We utilized a ribosomal profiling screen to identify messenger RNA transcripts actively translated in the spinal cord upon noxious stimulation that lead to pain-related behaviours in mice. This approach capitalizes on the finding that a structural component of the ribosome, the S6 protein, becomes phosphorylated—in both neuronal and non-neuronal cells—upon a wide variety of stimuli^11^. Using antibodies against the phospho-moiety of S6 (pS6), it is possible to biochemically isolate activated polysomes from tissue of stimulated animals and identify actively translated mRNAs by RNA sequencing (RNA-seq)^11–13^. Using anti-pS6 antibodies, we found that pain stimuli effectively induced S6 protein phosphorylation in the spinal cord of mice (Extended Data Fig. 1a–d). We tested several anti-pS6 antibodies and identified clone 5364 as the most effective antibody for biochemical isolation and enrichment of pain stimulation-activated polysomes from the mouse dorsal spinal cord (Extended Data Fig. 1e,f).

We expected that transcripts of the immediate early genes *cFos* and *FosB*, which are induced in the spinal cord upon pain stimulation^14^ (Extended Data Fig. 1b), would be enriched in biochemically isolated pS6 ribosome pull-downs, which was indeed the case (Extended Data Fig. 1g). Also, pS6 ribosomal profiling enriched cFos transcripts compared with total lysates obtained from the spinal cord of pain-stimulated mice, albeit only slightly (Extended Data Fig. 1h), suggesting that pS6 ribosomal profiling is a slightly more sensitive method to detect actively transcribed-translated genes compared with simply using spinal cord tissue for direct transcriptional profiling of spinal cord lysates.

**Fig. 1:**
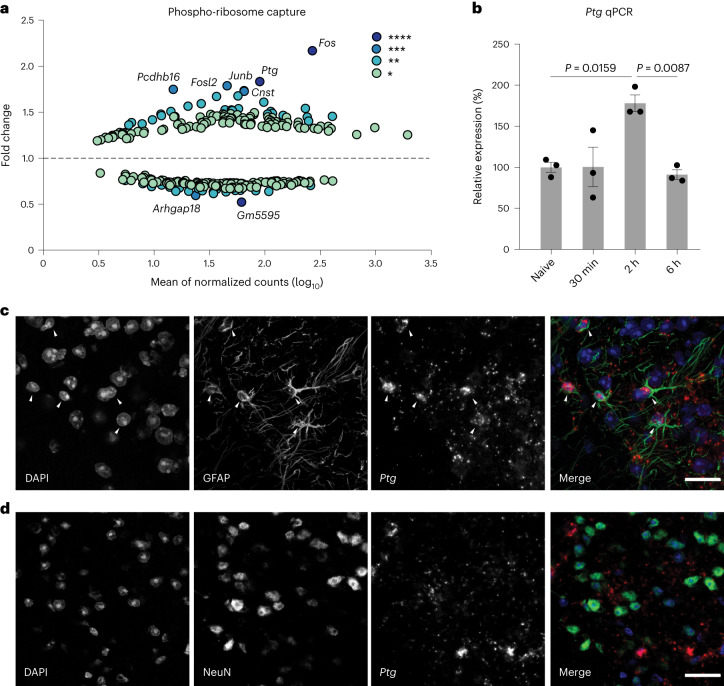
Noxious, pain-inducing stimulation triggers *Ptg* expression in astrocytes of the dorsal spinal cord. **a**, RNA-seq of pS6 captured ribosomes isolated from ipsilateral and contralateral spinal dorsal horn 2 h after mice had been subjected to FA stimulation. The *y* axis indicates relative enrichment or de-enrichment (ratio) of transcripts detected in ipsilateral (affected) dorsal horn tissue versus contralateral tissue. The *x* axis indicates relative mRNA abundance. Only significantly changed transcripts are shown (colour code indicates *P* value, *P < 0.05, **P < 0.01, ***P < 0.005, ****P < 0.001; two-tailed *t*-test; *n* = 3 pooled samples from 5 mice each). **b**, qPCR-determined relative expression level of the *Ptg* gene at different time points after FA stimulation (*n* = 3 mice). One-way ANOVA with Tukey’s post hoc test. **c**,**d**, Representative images of combined immunohistochemistry and fluorescence in situ hybridizations with antibodies directed against GFAP (**c**, green) and NeuN (**d**, green) and an in situ probe directed against *Ptg* (red) and DAPI (blue); scale bars, 20 µm; data represent mean ± s.e.m. See also Extended Data Fig. 1.

Injecting formalin (FA) into the intraplantar surface of the mouse paw is a widely used pain model^15^. When ipsilateral (the FA-stimulated side of the spinal cord) and contralateral (the non-FA-injected control side) spinal cord sections, corresponding to the relevant (hindpaw) dermatome levels lumbar 3 (L3)–L6, were compared by pS6 ribosomal profiling, we identified *Ptg* (Protein Targeting to Glycogen; also referred to as *Ppp1r3c*) as the most robustly induced gene, next to *cFos* (Fig. 1a and Extended Data Fig. 1a–d). Pain-induced *Ptg* expression was verified by quantitative PCR (qPCR) (Fig. 1b) and multi-colour in situ hybridization (Extended Data Fig. 1d), confirming that *Ptg* induction is confined to the pain stimulation-affected (ipsilateral) side of the dorsal spinal cord.

On the cellular level, we found that astrocytes—rather than neurons—express *Ptg* upon pain stimulation (Fig. 1c,d). Next to spinal astrocytes and neurons, spinal microglia have also been implicated in pathological forms of pain^16–18^. However, we did not find any *Ptg* expression in microglia in the context of noxious FA stimulation (Extended Data Fig. 1i).

### Noxious stimulation dynamically modulates spinal glycogen levels

PTG is a regulator of glucose and glycogen metabolism in that the molecule directs protein phosphatase 1 (PP1) to glycogen synthase and glycogen phosphorylase to promote dephosphorylation of these two key anabolic/catabolic glycogen enzymes, thereby inducing glycogen formation and inhibiting glycogen breakdown, respectively^19^.

Of note, in the brain glycogen is primarily stored in astrocytes^20–22^ and in vitro studies of brain-derived neuron–astrocyte cocultures show that forced expression of PTG in cultured astrocytes is sufficient to drive glycogen build-up^23^.

**Fig. 2:**
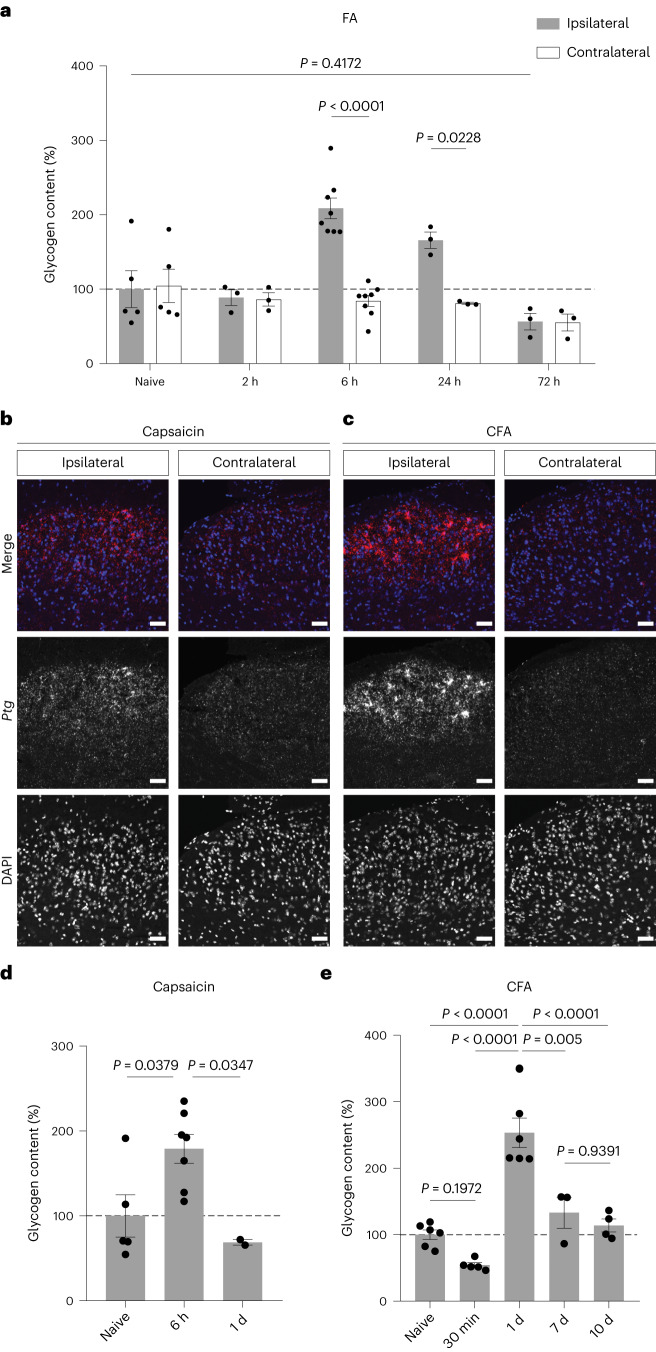
Inflammatory pain induces glycogen accumulation in the dorsal spinal cord. **a**, Glycogen content of ipsilateral and contralateral dorsal spinal cord tissue isolated at different time points after FA stimulation and expressed as percentage of glycogen of ipsilateral tissue isolated from naive mice (*n* = 6 for naive; *n* = 3 for 2 h; *n* = 8 for 6 h; *n* = 3 for 24 h and 72 h); two-way ANOVA with Bonferroni post hoc test. **b**,**c**, Representative images of in situ hybridizations (RNAscope) with a probe for *Ptg* (red) of spinal cord tissue 2 h after capsaicin stimulation (**b**) or CFA stimulation (**c**) and DAPI (blue); scale bars, 50 µm. **d**,**e**, Glycogen content (percentage of naive) of dorsal spinal cord tissue at different time points after capsaicin stimulation (**d**), with *n* = 5 mice for naive and *n* = 7 for 6 h and *n* = 2 for 1 d, or CFA stimulation (**e**), with *n* = 6 for naive, *n* = 5 for 30 min, *n* = 6 for 1d, *n* = 3 for 7 d and *n* = 4 for 10 d; one-way ANOVA with Tukey’s post hoc test. Data represent mean ± s.e.m. See also Extended Data Fig. 2.

We therefore wondered whether a noxious stimulus that induces pain would result in increased spinal glycogen levels. Indeed, glycogen levels increased on the ipsilateral but not on the contralateral side of the spinal cord subsequent to FA-induced pain (Fig. 2a). Interestingly, glycogen level increases were detectable with a delay of 6 h after the stimulus—4 h after *Ptg* mRNA peaked—and dropped to baseline levels within 1–3 d.

Next, we asked whether other types of painful stimuli would also trigger *Ptg* induction and protracted glycogen increases in the spinal cord. Similar to FA, stimulation with capsaicin or Complete Freund’s Adjuvant (CFA) induced *Ptg* mRNA expression on the ipsilateral side of the dorsal spinal cord (Fig. 2b,c). When assessing glycogen levels, we found that the shorter-acting capsaicin also induced glycogen elevation, albeit less pronounced compared with that of FA (Fig. 2d). On the other hand, CFA, a potent inducer of inflammatory pain^15,24,25^, triggered glycogen increases similar to those observed for the FA pain model (Fig. 2e). Additionally, when assessing glycogen levels shortly after a noxious, pain-inducing stimulus, it appeared that glycogen levels initially—and only transiently—decreased (Fig. 2e).

**Extended Data Fig. 2:**
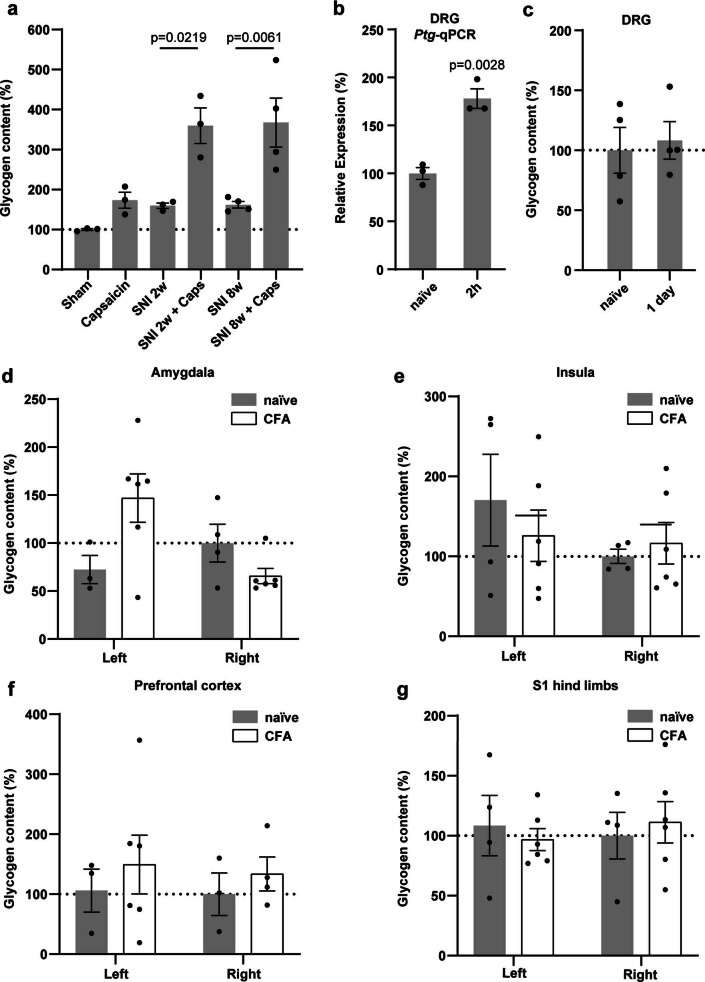
Different to the spinal cord, pain stimuli do not promote major glycogen accumulation in DRGs and higher brain centers processing pain signals (related to Fig. 2). **(a)** Glycogen content of ipsilateral dorsal spinal cord tissue compared to that of the contralateral side 2 or 8 weeks after Spared Nerve Injury- (SNI-)induced pain and with or without intraplantar capsaicin injection 6 h prior to tissue preparation; expressed as percent glycogen content of the ipsilateral dorsal spinal cord of sham/untreated mice. Note that SNI or capsaicin alone only slightly elevate glycogen levels while the combination of the two synergistically increases glycogen accumulation. (N = 3 mice); one-way ANOVA with Tukey’s post hoc test. **(b)** qPCR-determined relative expression level of *Ptg* mRNA transcripts in L2-L5 DRGs 2 hours after formalin stimulation compared to naïve DRGs (N = 4 mice). Unpaired two-tailed t-test. **(c)** Glycogen content of ipsilateral L2-L5 DRGs 1 day after formalin injection compared to that of naïve mice (N = 4 independent samples of 4 mice). **(d-g)** Glycogen content in the amygdala (**d**), insula cortex (**e**), prefrontal cortex (**f**) and hindlimb region of the somatosensory cortex (**g**) isolated from naïve or 6 h CFA-treated mice. Glycogen content of these bilateral brain regions is expressed as percent of the average of the left and right side of the naïve mice. Two-way ANOVA with Bonferroni post hoc test; data shown as mean ±s.e.m. N = 4 mice for naïve animals and N = 6 animals for CFA-treated animals.

These results suggest that a stronger and longer-lasting noxious stimulus resulted in stronger and longer-lasting glycogen build-up after the acute phase of the pain-inducing stimulus. We therefore also tested whether the combination of two disparate painful stimuli would result in additive glycogen accumulation. Spared-nerve injury (SNI), a model of neuropathic pain^26^, by itself only slightly increased glycogen levels, similar to a short-term capsaicin stimulation (Extended Data Fig. 2a). However, combing the two pain models resulted in synergistically increased spinal glycogen accumulation compared with mice subjected to either SNL or capsaicin treatment alone (Extended Data Fig. 2a).

Collectively, these results demonstrate that pronounced glycogen dynamics accompany diverse inflammatory pain stimuli in the spinal cord and appear to be graded, at least to some extent, by the intensity and duration of the noxious pain-inducing stimulus.

We wondered whether a pain-stimulated glycogen increase is specific to the spinal cord or whether other parts of the pain signalling pathway and distributed pain processing areas in the brain also show increased glycogen levels. The spinal cord receives signals from sensory neurons of the dorsal root ganglia (DRG), which contain satellite glia that resemble astrocytes in genetic make-up and functionality^27^. We therefore asked whether *Ptg* and glycogen induction can also be observed in DRGs. While *Ptg* induction is detectable in DRGs isolated from CFA-treated animals, glycogen was not markedly increased, at least not after 1 d, the time point when glycogen levels reach maximal levels in the spinal cord (Extended Data Fig. 2b,c). From the dorsal spinal cord, pain signals are relayed for further processing to higher-order brain centres. CFA-induced pain did not alter glycogen levels in the somatosensory, insula or prefrontal cortices or in the amygdala (Extended Data Fig. 2d–g), all of which brain regions are implicated in pain signal processing.

While we cannot exclude that small, cell-type-selective glycogen changes also occur in other parts of the nervous system involved in pain signal processing, strong and long-lasting painful insults appear to primarily modulate glycogen dynamics in the spinal cord.

### Noxious stimuli-induced spinal glycogen build-up is PTG dependent

**Fig. 3:**
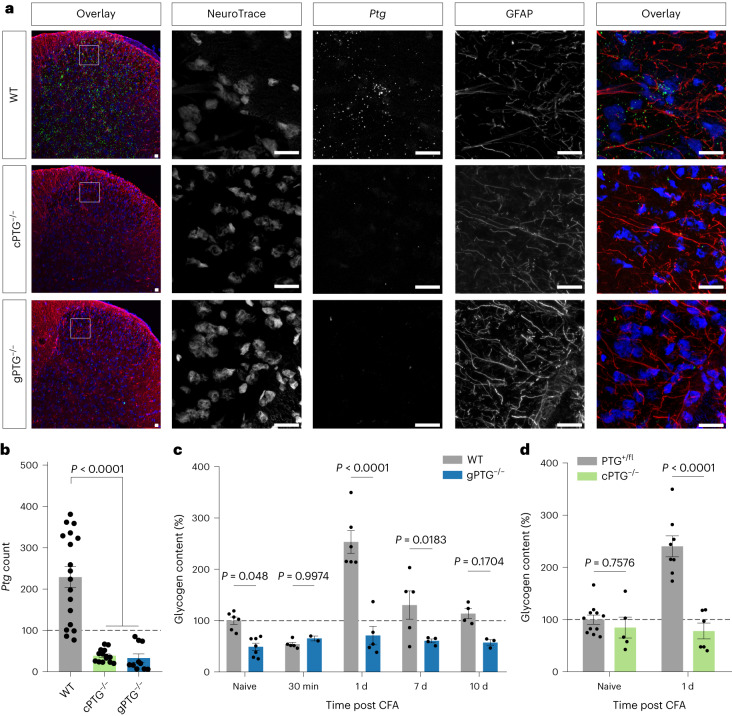
Noxious stimulation-induced spinal glycogen dynamics is blunted in PTG^−/−^ mice. **a**, Representative confocal images of in situ hybridizations (RNAscope) with a probe against *Ptg* (green) in the ipsilateral dorsal spinal cord tissue from WT, cPTG^−/−^ and gPTG^−/−^ mice 2 h after CFA-induced pain, and its colocalization with the neuronal marker (NeuroTrace) and an astrocytic marker (GFAP) in blue and red, respectively. Scale bars, 20 µm. **b**, Quantification of *Ptg* mRNA signals visualized by RNAscope in WT and PTG KO models as shown in **a** (quantified are the number of *Ptg* ‘dots’ in 3 images from *n* = 3 animals per genotype). **c**,**d**, Glycogen content (expressed as percentage of glycogen content measured in dorsal spinal cord tissue of naive WT control mice) at different time points after CFA stimulation of gPTG^−/−^ mice (**c**, *n* = 5 samples of 5 mice) and cPTG^−/−^ mice (**d**, *n* = 5 samples of 5 mice) compared with their respective control mice. Two-way ANOVA with Bonferroni post hoc test. Data represent mean ± s.e.m. See also Extended Data Fig. 3.

**Extended Data Fig. 3:**
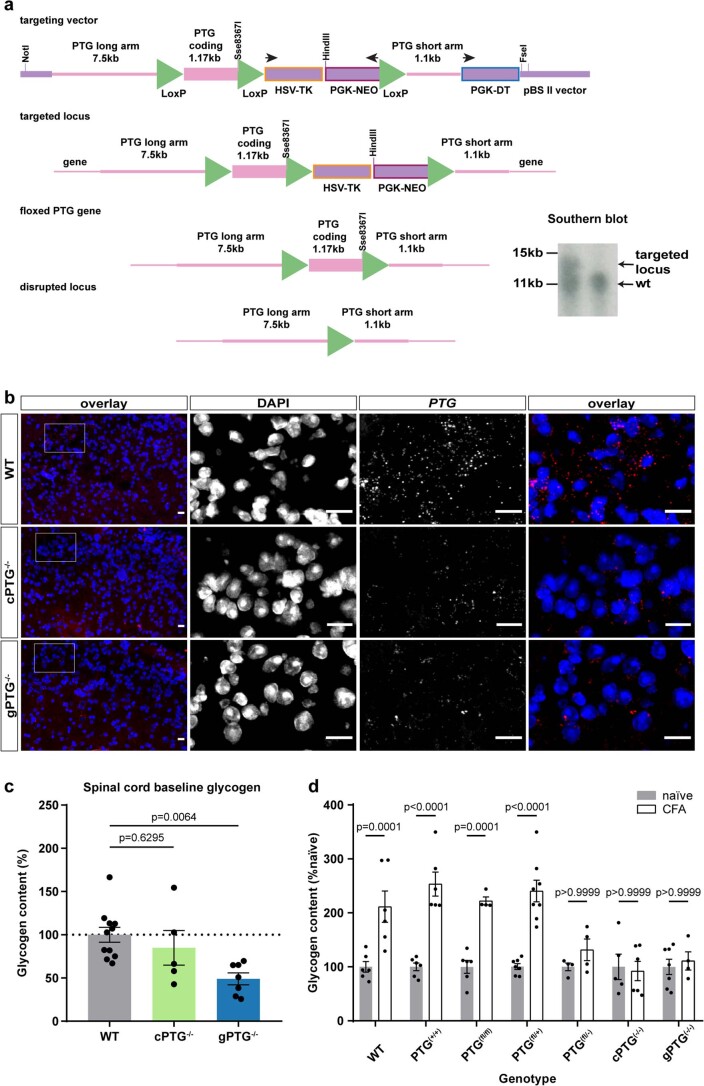
*Ptg* deletion blunts pain-induced glycogen build-up in the dorsal spinal cord (related to Fig. 3). **(a)** Embryonic stem cell targeting strategy to conditionally delete the single coding *Ptg* exon by introducing upstream and downstream loxP sites (green triangles). On the right panel a representative southern blot is shown, indicating the targeted locus by digesting genomic DNA with NcoI that cuts outside the two targeting arms resulting in a wildtype band of 11.5 kb and a targeted band of 13 kb. **(b)** Representative images of *in situ* hybridizations (RNAscope^®^) with a probe for *Ptg* (red) of ipsilateral dorsal spinal cord tissue from wildtype, cPTG^−/−^ and gPTG^−/−^ mice 2 hours after formalin stimulation. DAPI (blue), scale bar: 20 μm. **(c)** Glycogen content (percent of WT) of dorsal spinal cord tissue from the three indicated mouse lines without any stimulation (WT: N = 11; cPTG^−/−^:N = 5; gPTG^−/−^: N = 7); one way ANOVA with Tukey’s post hoc test; data represent mean ± s.e.m. **(d)** Glycogen content (percent of naïve) of dorsal spinal cord tissue from all the different mouse line genotypes used in this study. (WT (C57bl/6N mice): N = 6; PTG^+/+^: N = 6; PTG^fl/fl^: N = 4; PTG^+/fl^: N = 7; PTG^fl/−^: N = 4; cPTG^−/−^: N = 5; gPTG^−/−^: N = 4; Two-way ANOVA with Bonferroni’s post hoc test; data represent mean ± s.e.m.

As pain-induced glycogen increases followed *Ptg* mRNA expression with a delay of a few hours—allowing for the translation of PTG protein in due course—we reasoned that spinal glycogen build-up is dependent on PTG protein expression. We therefore generated conditional (floxed) PTG knockout (KO) animals (Extended Data Fig. 3a). We first crossed these conditional PTG KO mice with a Cre deleter mouse strain^28^ to delete functional PTG from the mouse germline and all tissues to obtain complete (global) PTG KO animals (gPTG^−/−^). We verified that pain-stimulated *Ptg* induction in spinal astrocytes was abolished in gPTG^−/−^ mice using multi-colour in situ hybridization (Fig. 3a,b and Extended Data Fig. 3b) and CFA-induced spinal glycogen increases were indeed blunted compared with wild-type (WT) control mice (Fig. 3c). Even baseline glycogen appeared to be reduced in gPTG^−/−^ mice to levels that were observed shortly after pain stimulation in WT mice (Fig. 3c and Extended Data Fig. 3c). To assess whether these effects are mediated by astrocytic PTG, we next crossed ‘floxed’ PTG mice to Aldh1L1-Cre^ERT2^ mice^29^ to ablate functional PTG from astrocytes by tamoxifen injections (we refer to these astrocytically *Ptg*-deleted mice from here on as cPTG^−/−^ mice). Side-by-side comparison of *Ptg* mRNA content in histological sections of WT and gPTG and cPTG KO animals demonstrated that *Ptg* is largely induced in spinal astrocytes (Fig. 3a,b and Extended Data Fig. 3b). However, these results do not rule out the possibility that baseline levels of *Ptg* are present in neuronal and/or other non-neuronal cells.

Similar to gPTG^−/−^ mice, in these astrocytically deleted cPTG^−/−^ mice, CFA pain-induced glycogen build-up was also completely blunted (Fig. 3d). Different from gPTG^−/−^ mice, however, baseline glycogen levels appeared normal in cPTG^−/−^ animals (Fig. 3d and Extended Data Fig. 3c,d), suggesting that non-astrocytic (yet PTG-dependent) glycogen stores may contribute to baseline spinal glycogen levels. Alternatively, it is also possible that the conditional KO approach did not affect baseline astrocyte glycogen levels within the timeframe of the tamoxifen-induced PTG deletion (starting 3 weeks before the experiment). Regardless of the origin of the low baseline glycogen levels, these results clearly demonstrate that astrocytic PTG is required for nociceptive glycogen dynamics.

### PTG deletion promotes faster recovery from inflammatory pain

**Fig. 4:**
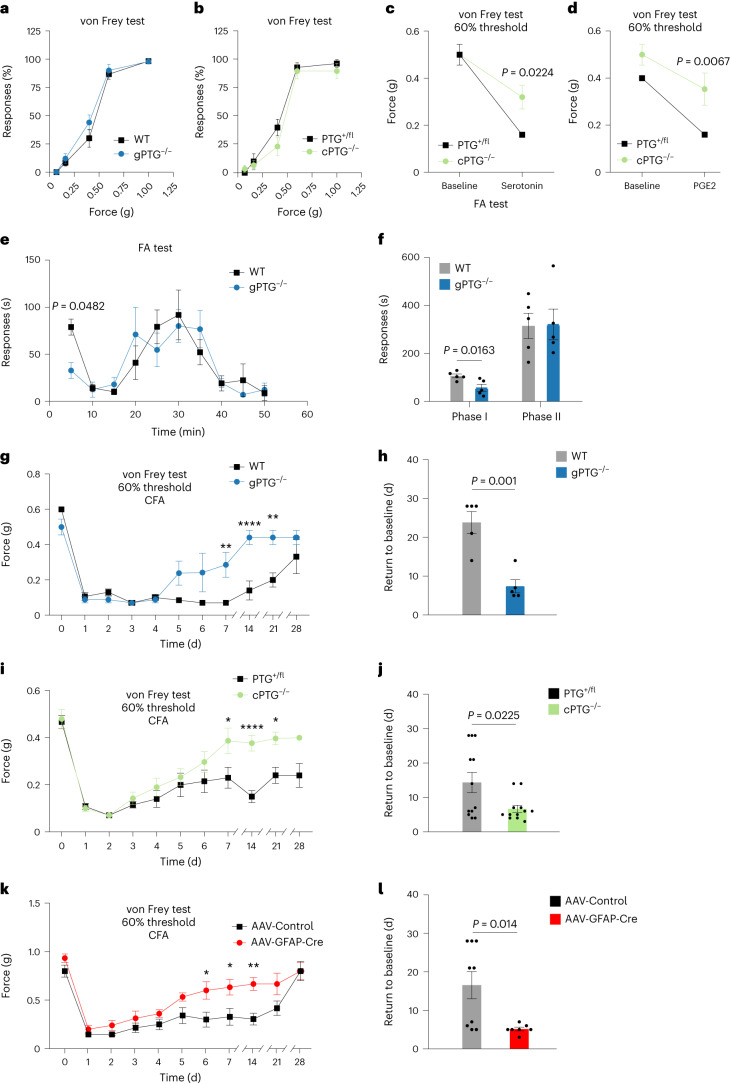
Inflammatory nociceptive sensitization and pain maintenance is reduced in PTG^−/−^ mice. **a**,**b**, Baseline mechanical sensitivity for WT and gPTG^−/−^ mice (**a**, *n* = 6) or PTG^+/fl^ and cPTG^−/−^ mice (**b**, *n* = 12). **c**,**d**, Mechanical threshold required to elicit a response in at least 60% of trials in PTG^+/fl^ and cPTG^−/−^ mice either not treated (baseline) or at 15 min after intraplantar serotonin (**c**, *n* = 6) or PGE2 (**d**, *n* = 6) injection into the hindpaw. **e**,**f**, Time course of FA-induced nocifensive responses scored in 5-min bins (**e**) or separated into Phase I (0–10 min) and Phase II (10–50 min) (**f**) for WT and gPTG^−/−^ mice (*n* = 5). **g**,**i**,**k**, The 60% mechanical threshold measured before and at different time points after CFA stimulation comparing WT (*n* = 5) and gPTG^−/−^ mice (**g**, for 7 d **P = 0.0092; for 14 d ****P < 0.0001; for 21 d **P = 0.0025; *n* = 5), PTG^+/fl^ and cPTG^−/−^ mice (**i**, for 7 d *P = 0.013; for 14 d ****P < 0.0001; for 21 d *P = 0.013; *n* = 12), and AAV-Control- (pAAV.GFAP.eGFP.WPRE.hGH, *n* = 9) and AAV-GFAP-Cre- (pssAAV-2-hGFAP-mCherry_iCre-WPRE-hGH, *n* = 7) injected PTG^fl/fl^ mice (**k**, for 6 d *P = 0.0252; for 7 d *P = 0.0216; for 14 d **P = 0.0048). **h**,**j**,**l**, Number of days required to recover from a sensitized state back to baseline mechanical threshold (cutoff: 1 s.d. of the average baseline value) after intraplantar CFA injection of WT and gPTG^−/−^ mice (**h**), PTG^+/fl^ and cPTG^−/−^ mice (**j**), and AAV-Control- and AAV-GFAP-Cre-injected PTG^fl/fl^ mice (**l**). Data correspond to traces shown in **g**, **i** and **k**. **a**–**g**, **i** and **k**, two-way ANOVA with Bonferroni post hoc test; **h**, **j** and **l**, unpaired two-tailed *t*-test. Data represent mean ± s.e.m. In all behaviour experiments littermates were used as controls. See also Extended Data Fig. 4.

**Extended Data Fig. 4:**
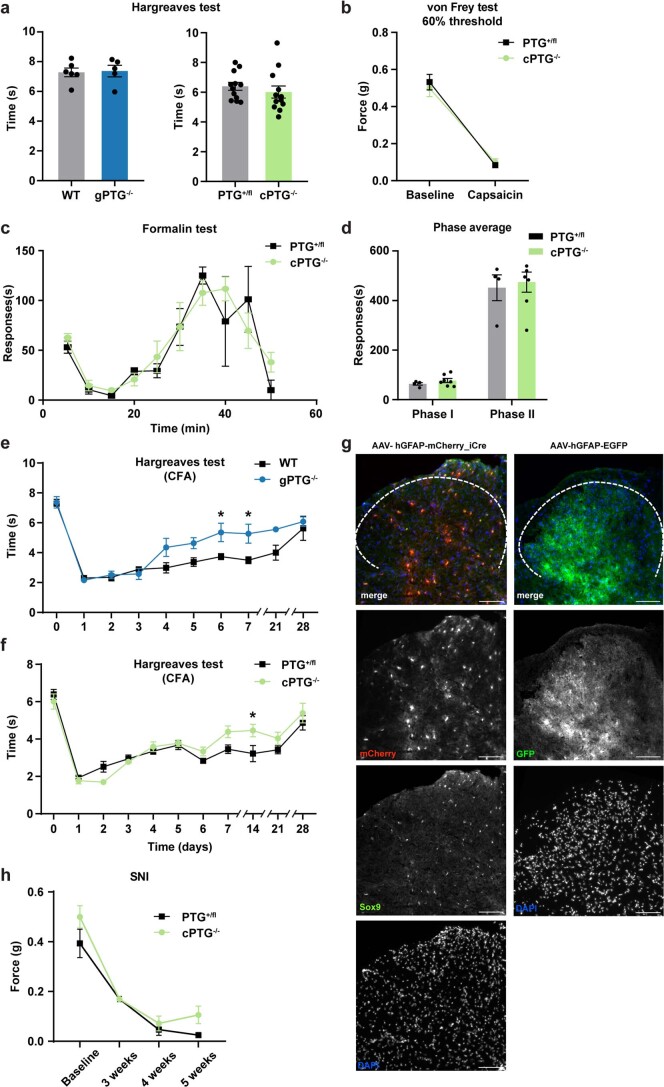
Acute pain is largely unperturbed in PTG^−/−^ animals (related to Fig. 4). **(a)** Acute nocifensive responses upon heat stimulation using a focal light source directed onto the hindpaw of mice (Hargreaves test). Response latency was similar in cPTG^−/−^, gPTG^−/−^ and their respective wildtype littermate or cPTG^+/fl^ control mice (WT: N = 6; gPTG^−/−^, PTG^+/fl^: N = 12; cPTG^−/−^: N = 12); unpaired two-tailed t-test. **(b)** Mechanical threshold required to elicit a response in at least 60% of trials in PTG^+/fl^ and cPTG^−/−^ mice that are either not treated (baseline) or 15 minutes after intraplantar capsaicin injection into the hindpaw (N = 6); two-way ANOVA with Bonferroni post hoc test. **(c**, **d)** Time course of formalin-induced nocifensive responses scored in 5 min bins (**c**) or separated in Phase I (0-10 min) and Phase II (10-50 min) (**d**) for cPTG^−/−^ mice (N = 4) and cPTG^+/fl^ control mice (N = 7). Two-way ANOVA with Bonferroni post hoc test. **(e**, **f)** Nocifensive responses upon heat stimulation using a focal light source directed onto the hindpaw (Hargreaves test) measured before and at different time points after CFA-induced pain comparing gPTG^−/−^ and WT littermate mice (**e**, for 6 days *p = 0.0496; for 7 days *p = 0.0210; N = 5) and cPTG^−/−^ and control PTG^+/fl^ mice (**f**, for 14 days *p = 0.0102; N = 12). Two-way ANOVA with Bonferroni post hoc test; data represent mean ± s.e.m. **(g)** Immunohistochemical analysis to visually corroborate virus expression in cryosections of the spinal cord. Left panels show colocalization of GFAP driven expression of iCre fused to mCherry and Sox9, a nuclear astrocyte marker. Right panels show the expression of GFAP driven EGFP that was injected in the control mouse group. Note that mCherry expression is expected to be localized in the nucleus while EGFP is expected to fill the whole astrocytic cytoplasmic compartment. Scale bar: 20μm. **(h)** 60% mechanical threshold measured before and at different time points after sciatic nerve injury (SNI), comparing PTG^+/fl^ and cPTG^−/−^ mice (N = 6). Data represent mean ± s.e.m.

Given the dependence of noxious stimuli-induced spinal glycogen metabolism on PTG, we wondered if perturbation of astrocytic glycogen dynamics by deleting PTG would have any effect on behavioural pain-related responses in mice. In assessing pain-related behaviours, the measurement of the threshold and latency to (painful) mechanical and thermal stimuli applied to the animal’s hindpaw are standard pain testing paradigms^30^. We found that, under basal conditions, mechanical stimuli generated by applying von Frey filaments of different mechanical strength to the plantar hindpaw surface evoked normal responses in both gPTG^−/−^ and cPTG^−/−^ mice as compared with WT animals (Fig. 4a,b). Similarly, global or conditional PTG deletion did not affect the latency of heat-pain-triggered paw withdrawal when a light beam was focused on the plantar surface of the hindpaw (Hargreaves test; Extended Data Fig. 4a). We next evaluated whether PTG deletion would influence short-term pain sensitization such as that triggered by the intraplantar injection of capsaicin, serotonin, prostaglandin E2 (PGE2) or FA. We found that capsaicin provoked similar mechanical hypersensitivity in WT and cPTG^−/−^ mice (Extended Data Fig. 4b). Only inflammatory stimuli mediating longer-lasting pain sensitization, such as serotonin or PGE2 (ref. ^31^), were associated with reduced mechanical hypersensitivity in cPTG^−/−^ mice (Fig. 4c,d). FA injection, which evokes spontaneous nocifensive responses such as licking, flinching and guarding of the affected paw, produced a slightly smaller response in the gPTG^−/−^ mice in the initial phase of the biphasic pain response. This difference appeared to be absent from cPTG^−/−^ mice, suggesting that intact baseline glycogen levels may be relevant for the early acute phase of FA-evoked nocifensive pain behaviour (Fig. 4e,f and Extended Data Fig. 4c,d). Importantly, the more pronounced second phase of the FA-triggered response was indistinguishable between WT and both KO models (Fig. 4e,f and Extended Data Fig. 4c,d).

Collectively, these data suggest that spinal glycogen levels and their utilization have only a small effect on acute noxious signal processing and pain-related rodent behaviours. For the most part, short-term acute pain signalling, such as that initiated by capsaicin, appeared not to depend on glycogen turnover and was not altered in the absence of the *Ptg* gene.

The absence of a strong phenotype related to acute pain signal processing in PTG^−/−^ animals is not entirely surprising given the observed delay to PTG induction and glycogen build-up subsequent to an inflammatory pain stimulus compared with the duration of short-term pain sensitization.

We therefore considered whether pathological long-term inflammatory sensitization and behaviour would be altered in PTG^−/−^ mice. Intraplantar CFA injection, which we found to generate strong induction in PTG-dependent spinal glycogen accumulation (Fig. 3c), sensitizes mice by lowering pain thresholds for up to 4 weeks^24,25^. As expected, both gPTG^−/−^ and cPTG^−/−^ animals became sensitized to mechanical and thermal stimuli similarly to the WT controls. Strikingly, however, gPTG^−/−^ and cPTG^−/−^ animals recovered faster from the sensitized state, reaching baseline pain thresholds already after 7 ± 2 d, as compared with WT mice, which remained sensitized for up to 24 ± 3 d (Fig. 4g–j and Extended Data Fig. 4e,f). In both PTG KO models, this accelerated recovery was observed for both mechanical- and heat-pain hypersensitivity, with a more pronounced beneficial effect on mechanical hypersensitivity.

We noted that pain-induced glycogen increases were reduced already in heterozygous KO animals, bearing one globally deleted and one ‘floxed’ PTG allele (cPTG^−/fl^), suggesting that heterozygous KO mice may act as functional PTG hypomorphs upon pain stimulation but not under basal conditions (Extended Data Fig. 3d). Due to the *Ptg* ‘gene dosage’ effect and to verify whether the reduced inflammatory pain maintenance period observed in gPTG^−/−^ and cPTG^−/−^ mice was mediated by astrocytic glycogen dynamics, we infected PTG^fl/fl^ mice in lumbar spinal regions L2–L6 with adeno-associated virus (AAV) particles delivering Cre in a GFAP-dependent (astrocyte-specific) manner. Indeed, deleting the *Ptg* gene specifically in astrocytes of adult mice recapitulated the phenotype of global and conditional KO mice and allowed the animals to recover faster from CFA-induced inflammatory pain compared with control AAV-injected animals (Fig. 4k,l and Extended Data Fig. 4g).

We also tested whether astrocytic PTG and glycogen dynamics modulate mechanical hypersensitivity in the SNI model of neuropathic pain. When subjecting cPTG^−/−^ animals and littermate controls to SNI, we did not observe a difference between the two animal groups in mechanical threshold responses tested up to 5 weeks after SNI (Extended Data Fig. 4h). This outcome is reminiscent of previous results emphasizing a role of spinal astrocytes in inflammatory pain but a lesser contribution to SNI-related pain behaviour^8^.

In summary, our data demonstrate that, while short-lasting sensitization is largely unaffected in PTG^−/−^ mice, recovery from strong and long-lasting inflammatory pain is accelerated when astrocytic glycogen induction is blunted.

### Noxious stimuli-induced glycolytic capacity is PTG dependent

Neuronal transmission, signal processing and plasticity require energetic metabolic support. Neurons receive energetic metabolites such as glucose via the bloodstream. However, upon increased demand, for example, in the context of plasticity-triggering synaptic activity, it has been shown that neurons may depend on additional metabolic fuel from astrocytes^32–39^. We therefore speculated whether inflammatory signal processing leading to pain may modulate spinal energy consumption and, more importantly, whether astrocytic glycogen stores are a relevant fuel source.

To measure energy consumption and respiration in the spinal network, we utilized the so-called Seahorse assay using mouse spinal cord slice preparations. This assay enables measurements of the rates of glycolysis (assessed by the extracellular acidification rate) and mitochondrial respiration (assessed by the oxygen consumption rate (OCR))^40^. To the best of our knowledge, this assay has not been described for spinal cord tissue before. Therefore, we first established spinal slice preparations and optimized measurement conditions (see the Methods section for details), based on a protocol established for brain slices^41^.

**Extended Data Fig. 5:**
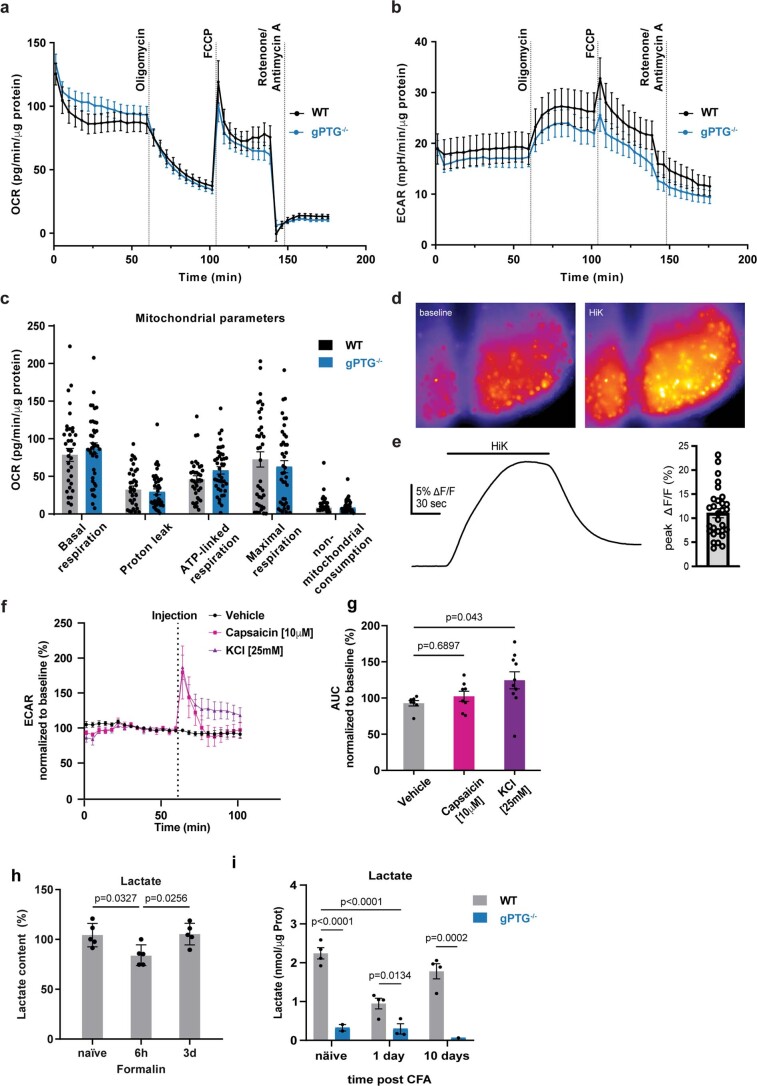
Basic spinal respiratory parameters and lactate abundance in the absence of PTG (related to Fig. 5). **(a**-**c)** Seahorse mitochondrial-stress test in spinal cord dorsal horn slices comparing Oxygen Consumption Rate OCR **(a)**, Extracellular Acidification Rate ECAR **(b)** and mitochondrial parameters **(c)** between WT (N = 37 slices/5 mice) and gPTG^−/−^ (N = 40/5). **(d)** Representative images of the neuronally-expressed calcium indicator jRGECO1a.NLS.Flag showing fluorescence prior to (baseline) and during (HiK) stimulation with high-potassium (25 mM) aCSF. **(e)** Representative trace showing the percent change in fluorescence intensity over the course of the imaging period within a large region of interest encompassing all visibly labeled cells within the dorsal horn (left) and peak amplitudes of nuclear calcium responses measured within 2 minutes after the start of stimulation in 29 slices from N = 4 animals (right). **(f)** Effect of 10 μM capsaicin and 25 mM KCl on ECAR in WT spinal cord dorsal horn slices. Data normalized to the average baseline of each condition (N = 7/1). **(g)** Change of ECAR produced by the stimulus (capsaicin or KCl) measured as change of area under the curve (AUC) derived from data shown in (**f**; One-way ANOVA N = 7/1). **(h)** Lactate content after formalin pain stimulus, measured by mass spectrometry and normalized to naïve animals. One-way ANOVA with Tukey’s post hoc test (N = 5). **(i)** Lactate content in spinal cord dorsal horn tissue of WT and gPTG^−/−^ mice before and at indicated time points after CFA injection. Two-way ANOVA (N = 4). Data represent mean ± s.e.m.

Next, we compared basic respiratory parameters in gPTG^−/−^ animals and WT controls. We did not find any significant differences in spinal cord tissue from these two groups of mice (Extended Data Fig. 5a–c). This is in agreement with results showing that astrocytic PTG and glycogen appear not to be required for basic spinal cord function because basal mechanical and thermal sensitivity are normal in gPTG^−/−^ animals (Fig. 4a,b and Extended Data Fig. 4a,b).

We next assessed whether mimicking painful stimulation by increasing neuronal activity (action potential (AP) firing), either by supplying the TRPV1 agonist capsaicin or by perfusing spinal slices with high potassium chloride (KCl) (Extended Data Fig. 5d,e), would trigger increased energy consumption. Indeed, both paradigms resulted in increased glycolytic activity in spinal slices obtained from WT mice, with KCl provoking a more robust response (Extended Data Fig. 5f,g).

Since we found CFA-induced inflammatory pain to trigger strong glycogen dynamics that were completely blunted in PTG^−/−^ mice, and because PTG^−/−^ mice recovered faster than WT littermates from CFA-evoked long-lasting pain hypersensitivity, we reasoned that neuronal activation on the background of CFA-induced pain would potentially reveal altered energy consumption in spinal slices of gPTG^−/−^ mice.

**Fig. 5:**
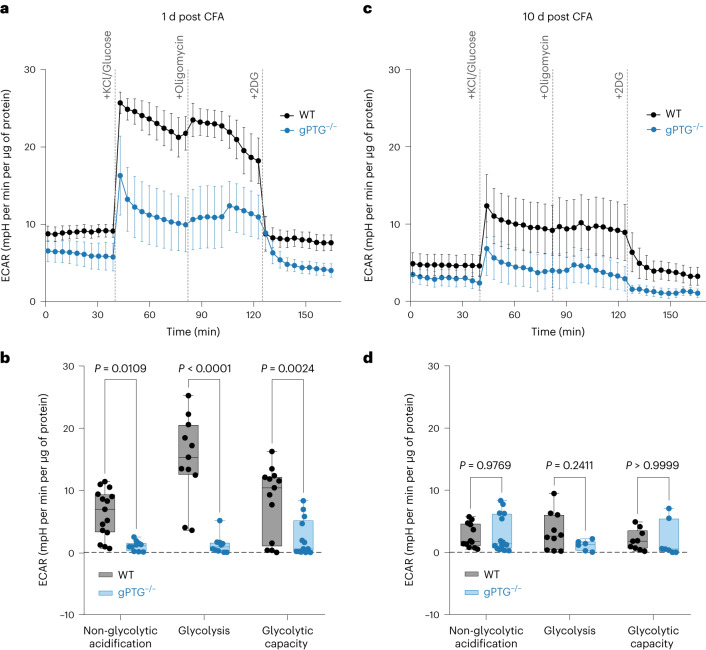
Inflammatory pain-induced glycolytic capacity in spinal cord circuits is blunted in the absence of PTG. **a**,**c**, Representative curves of Seahorse glycolytic rate assay during neuron stimulation induced by 25 mM KCl in spinal dorsal horn slices collected 1 d (**a**) and 10 d post CFA stimulation (**c**) of WT (*n* = 7 samples in *n* = 3 mice (*n* = 7/3) for 1 d, *n* = 10/3 for 10 d) and gPTG^−/−^ mice (*n* = 5/3 for 1 d and *n* = 10/3 for 10 d). **b**,**d**, Comparison of non-glycolytic acidification, glycolysis and glycolytic capacity at 1 d (**b**) and 10 d (**d**) post CFA stimulation of WT mice (*n* = 12/3 for 1 d, *n* = 7/3 for 10 d) and gPTG^−/−^ mice (*n* = 10/3 for 1 d and *n* = 10/3 for 10 d). Two-way ANOVA with Sidak’s post hoc test. Data represent mean ± s.e.m. Boxes extend from the 25th to 75th percentiles; whiskers indicate smallest and largest values; centre lines represent median. See also Extended Data Fig. 5. 2DG, 2-deoxy-d-glucose; ECAR, extracellular acidification rate; mpH, milli-pH.

We therefore determined glycolytic capacity in spinal cord slices of WT and gPTG^−/−^ mice at 1 d and 10 d after intraplantar CFA injection. Interestingly, the glycolytic response upon neuronal (KCl) activation was reduced in gPTG^−/−^ spinal tissue slices obtained from animals 1 d after CFA treatment as compared with the WT controls (Fig. 5a,b). These results suggest that glycolytic capacity in the dorsal spinal cord during inflammatory pain requires elevated astrocytic glycogen stores. On the other hand, 10 d after the induction of CFA-triggered inflammatory pain hypersensitivity, glycolytic capacity was reduced for both groups of mice and appeared slightly—albeit not significantly—different between the two genotypes (Fig. 5c,d). These differences in glycolytic capacity correlated with the differences in glycogen levels which were found to be robustly increased in WT (but not in gPTG^−/−^) mice 1 d after CFA injection and which had returned to baseline levels several days later (Fig. 3c).

As spinal glycolytic capacity was reduced in gPTG^−/−^ animals, we next determined whether levels of lactate, a downstream product of glycolysis, would also be changed. We found spinal lactate levels to be reduced in gPTG^−/−^ mice. Additionally, lactate levels in WT mice—but not in gPTG^−/−^ mice—were dynamically altered in spinal cord tissue upon pain stimulation (Extended Data Fig. 5h,i).

Taken together, these data support the hypothesis that spinal astrocytic glycogen dynamics evoked by robust noxious stimulation and signal processing promote astrocytes’ glycolytic capacity and lactate production, thereby prolonging pain maintenance.

### Spinal neuronal plasticity is fuelled by astrocytic glycogen

How could a deficit in the energy landscape of the spinal astrocyte–neuron network be beneficial to the recovery of long-lasting pain? Persistent noxious signalling, arising, for example, as a consequence of tissue damage, cancer or other forms of disease, can lead to structural and functional plasticity in the spinal network, resulting in increased sensitivity and activity of spinal pain circuits, culminating in increased sensitivity to peripheral stimuli^42–45^. We hypothesized that, similar to plasticity mechanisms found in the brain^39,46^, induction and maintenance of maladaptive pain-induced spinal plasticity requires metabolic energy that—at least in part—comes from astrocytes. To analyse spinal plasticity, which is associated with heightened synaptic activity and a presumably increased propensity of spinal projection neurons to fire APs^47^, we first measured changes in neuronal excitability in acute spinal slice preparations of WT mice to gauge whether this intrinsic neuronal property is enhanced in our CFA model of inflammatory pain and could serve as a measure of spinal plasticity.

**Fig. 6:**
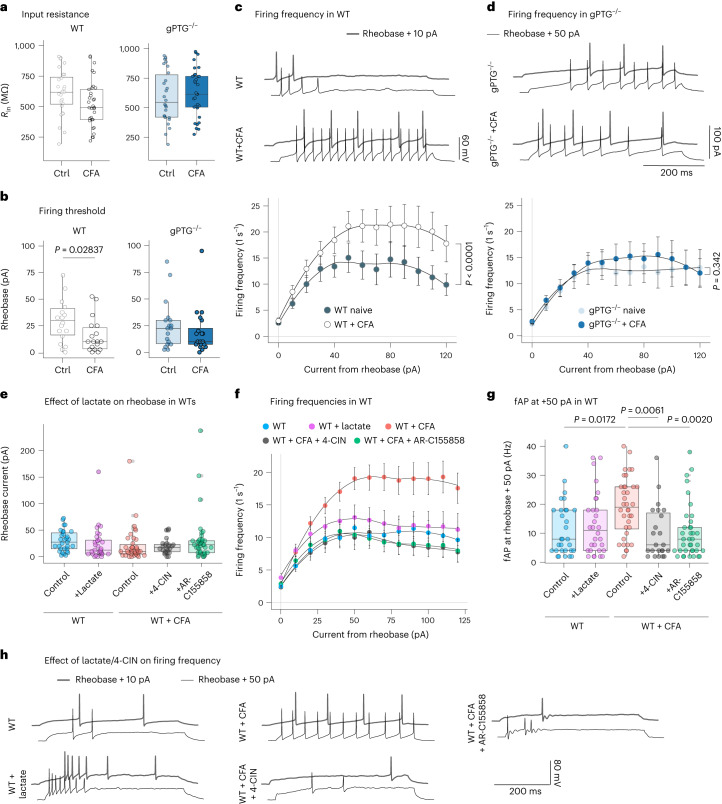
CFA-induced spinal neuronal plasticity is blunted in gPTG^−/−^ mice. **a**, Comparison of membrane *R*_in_ of randomly recorded L1 spinal neurons derived from naive and CFA-injected (10 d postinjection) WT and gPTG^−/−^ KO mice. *n* = 28 cells/4 animals (WT), *n* = 33/4 (WT + CFA), *n* = 26/4 (KO) and *n* = 33/4 (KO + CFA). **b**, Comparison of the rheobase in L1 neurons from naive and CFA-treated WT and gPTG^−/−^mice. *n* = 18/4 (WT), *n* = 18/4 (WT + CFA), *n* = 18/4 (gPTG^−/−^) and *n* = 20/4 (KO + CFA). Unpaired two-tailed *t*-test. **c**, Top: example traces of firing patterns in L1 neurons derived from WT naive and CFA-treated mice. Bottom: comparison of the firing frequencies across a range of 500 ms-long current injections (from 0 pA to 120 pA above rheobase) in WT and WT + CFA groups. *n* = 18/4 (WT) and *n* = 20/4 (WT + CFA). Two-way ANOVA (effect of CFA treatment). **d**, Top: example traces of firing patterns in L1 neurons derived from gPTG^−/−^ naive and CFA-treated mice. Bottom: comparison of the firing frequencies across a range of 500 ms-long current injections (from 0 pA to 120 pA above rheobase) in gPTG^−/−^ and gPTG^−/−^ + CFA groups. *n* = 18/4 (gPTG^−/−^) and *n* = 20/4 (gPTG^−/−^ + CFA). **e**, Rheobase comparison of WT/WT + CFA groups recorded after the addition of 4-CIN (300 μM), AR-C155858 (2 μM) or lactate (15 mM) into recording solution (aCSF). *n* = 31/4 (WT), *n* = 31/3 (WT + lactate), *n* = 38/4 (WT + CFA), *n* = 24/3 (WT + CFA + 4-CIN) and *n* = 30/3 (WT + CFA + AR-C155858). **f**, Comparison of the firing frequencies in response to 500 ms current injections (from 0 pA to 120 pA above rheobase) in naive/CFA WT groups in the presence of l-lactate, 4-CIN or AR-C155858. **g**, Comparison of firing frequencies at 50 pA above rheobase (based on **f**); *n* = 30/4 (WT), *n* = 31/3 (WT + lactate), *n* = 38/4 (WT + CFA), *n* = 24/3 (WT + CFA + 4-CIN) and *n* = 30/3 (WT + CFA + AR-C155858). One-way ANOVA, Sidak’s post hoc test (WT + CFA/WT + CFA + 4-CIN/WT + CFA + AR-C155858). **h**, Example traces of firing patterns of WT/WT + CFA neurons recorded in the presence of 4-CIN, AR-C155858 or lactate. Data are shown as mean ± s.e.m. Boxes in panels **a**, **b**, **e** and **g** extend from the 25th to 75th percentiles; whiskers indicate smallest and largest values; centre lines represent median. See also Extended Data Fig. 6. Ctrl, control; fAP, action potential frequency.

Therefore, we first measured the input resistance (*R*_in_) and the rheobase of randomly sampled L1 spinal neurons. The first parameter, *R*_in_, was not changed in neurons recorded from animals 10 d after CFA injection compared with naive control animals, suggesting that basic electrical plasma membrane properties of the neurons, such as the density of passive, non-voltage-dependent ‘leak’ channels, did not change as a consequence of CFA treatment (Fig. 6a). Based on previous work, we expected the excitability of WT L1 neurons to increase following inflammatory pain^47^. Indeed, we found that the minimum (rheobase) current necessary to elicit an AP during a 500 ms current injection step was significantly lower in CFA-treated WT neurons compared with the naive WT group (Fig. 6b), establishing this cell-intrinsic electrophysiological parameter as a bona fide inflammatory pain-induced spinal plasticity indicator.

Strikingly, CFA treatment did not affect the rheobase in gPTG^−/−^ neurons 10 d after intraplantar injection (Fig. 6b), suggesting that blunted glycogen dynamics prevented hyperexcitability. We further analysed the neuronal excitability of L1 neurons by injecting a range of depolarizing currents above the threshold (rheobase) current to evoke trains of APs. Again, we found that CFA treatment resulted in enhanced AP firing to current injections in WT neurons (Fig. 6c) but not in neurons derived from gPTG^−/−^ animals (Fig. 6d).

Our functional metabolic analysis suggested that lactate, a downstream metabolite of glycogenolysis/glycolysis, might play a role in mediating hyperexcitability. We therefore set out to test whether the firing properties of L1 spinal neurons can be modulated either by application of α-cyano-4-hydroxycinnamate (4-CIN; 300 µM), an inhibitor of monocarboxylate transporters (MCTs) involved in shuttling lactate between astrocytes and neurons^48^, or by supplementation of lactate in the perfusion fluid. 4-CIN did not affect rheobase in L1 neurons from either WT naive mice or WT mice at 10 d post CFA injection (Fig. 6e). Interestingly, however, the drug robustly reduced CFA-induced hyperexcitability in WT L1 neurons back to baseline levels when neurons were stimulated to fire trains of APs (Fig. 6f–h). Because 4-CIN is also known to inhibit the mitochondrial pyruvate carrier (MPC)^49^, we tested a chemically orthogonal drug (AR-C155858, 2 μM) that also targets MCTs but spares MPC^50^. Similarly, the AR compound replicated the effect of 4-CIN and reduced CFA-induced hyperexcitability of spinal neurons (Fig. 6f–h). Conversely, lactate, at concentrations shown to enhance firing activity of cortical neurons (15 mM)^46^, did not have any stimulatory effect on the firing properties of naive (non-CFA-treated) WT neurons (Fig. 6f–h), suggesting that lactate alone is insufficient to transform L1 neurons into a hyperexcitable state.

**Extended Data Fig. 6:**
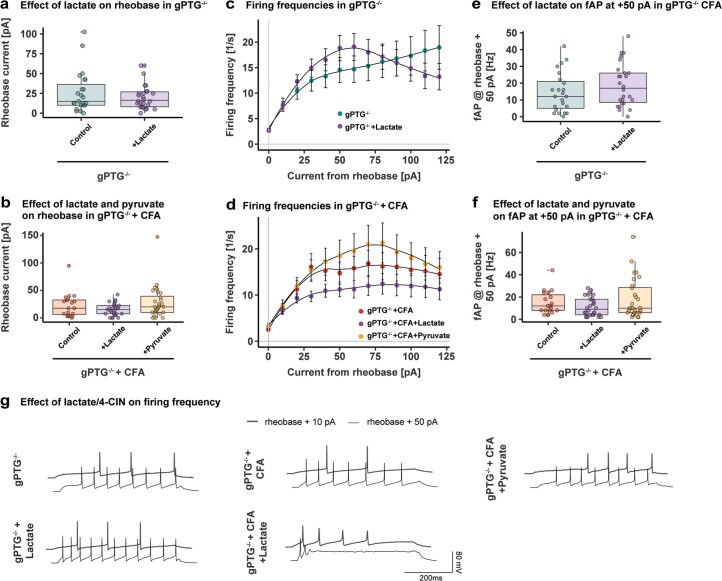
Lactate is not sufficient to increase excitability of spinal L1 neurons in the absence of PTG (related to Fig. 6). **(a)** Rheobase comparison in naïve gPTG^−/−^ neurons recorded with the addition of lactate (15 mM) into recording aCSF. N = 23 cells/N = 4 animals (gPTG^−/−^) and N = 26/3 (gPTG^−/−^ + Lactate). **(b)** Rheobase comparison in CFA-treated gPTG^−/−^ group recorded with the addition of lactate (15 mM) or pyruvate (15 mM) into recording aCSF. N = 19/4 (gPTG^−/−^+CFA), N = 28/3 (gPTG^−/−^+CFA+Lactate) and N = 28/3 (gPTG^−/−^+CFA+Pyruvate). **(c)** Comparison of the firing frequencies in response to 500 ms current injections (from 0pA to 120pA above rheobase) in naïve gPTG^−/−^ neurons in the presence of L-lactate. N = 23/4 (gPTG^−/−^) and N = 26/3 (gPTG^−/−^ + Lactate). **(d)** Comparison of the firing frequencies in response to 500 ms current injections (from 0pA to 120pA above rheobase) in CFA gPTG^−/−^ neurons in the presence of L-lactate or pyruvate. N = 19/4 (gPTG^−/−^+CFA), N = 28/3 (gPTG^−/−^+CFA+Lactate) and N = 28/3 (gPTG^−/−^+CFA+Pyruvate). **(e)** Comparison of firing frequencies at 50pA above rheobase current (based on **b**). N = 23/4 (gPTG^−/−^) and N = 26/3 (gPTG^−/−^ + Lactate). **(f)** Comparison of firing frequencies at 50pA above rheobase current (based on **e**). N = 19/4 (gPTG^−/−^+CFA), N = 28/3 (gPTG^−/−^+CFA+Lactate) and N = 28/3 (gPTG^−/−^+CFA+Pyruvate). **(g)** Example traces of firing patterns of gPTG^−/−^/gPTG^−/−^+CFA neurons recorded in the presence of lactate and pyruvate. Data shown as mean ± s.e.m. Boxes in panels a, b, e and f extend from the 25^th^ to 75^th^ percentiles, whiskers indicate smallest and largest values, center lines represent median.

Since we found L1 neurons from gPTG^−/−^ animals to be compromised in increasing AP firing rates following CFA treatment, we assessed whether adding lactate to gPTG^−/−^ mouse-derived spinal slices would be sufficient to increase their excitability. However, again lactate alone did not affect rheobase or hyperexcitability in gPTG^−/−^ slices, from either naive or CFA-treated animals (Extended Data Fig. 6). It has also been shown that lactate can decrease neuronal activity through Hydroxycarboxylic Acid Receptor 1 (HCAR1)^51^. We therefore tested whether the related molecule pyruvate, which does not activate this receptor^52^ but is able to enter cells via MCT transporters and can increase excitability in neurons^46^, was able to increase AP firing rates in gPTG^−/−^ slices. We found a subtle trend of increased excitability in gPTG^−/−^ neurons treated with pyruvate; however, when comparing all three (control, lactate and pyruvate treated) groups we found no significant difference (Extended Data Fig. 6d,f).

Together, these results suggest that astrocytic glycogen dynamics and glycolysis are important factors driving hyperexcitability of dorsal spinal L1 neurons in the context of inflammatory pain. However, acutely supplementing glycolytic products such as lactate or pyruvate 10 d after spinal plasticity had been triggered by CFA treatment did not significantly promote hyperexcitability in PTG^−/−^ mice.

### Several spinal metabolic pathways are modulated by PTG

Because we had found that PTG-regulated astrocytic glycogen stores were important for pain-induced (1) glycolytic capacity, (2) spinal hyperexcitability and, ultimately, (3) pain-related behaviour, we next considered whether these alterations were also reflected in changes of metabolic pathways and metabolite transporters, focusing on transcriptional analysis and measuring the activity of key regulatory enzymes.

**Extended Data Fig. 7:**
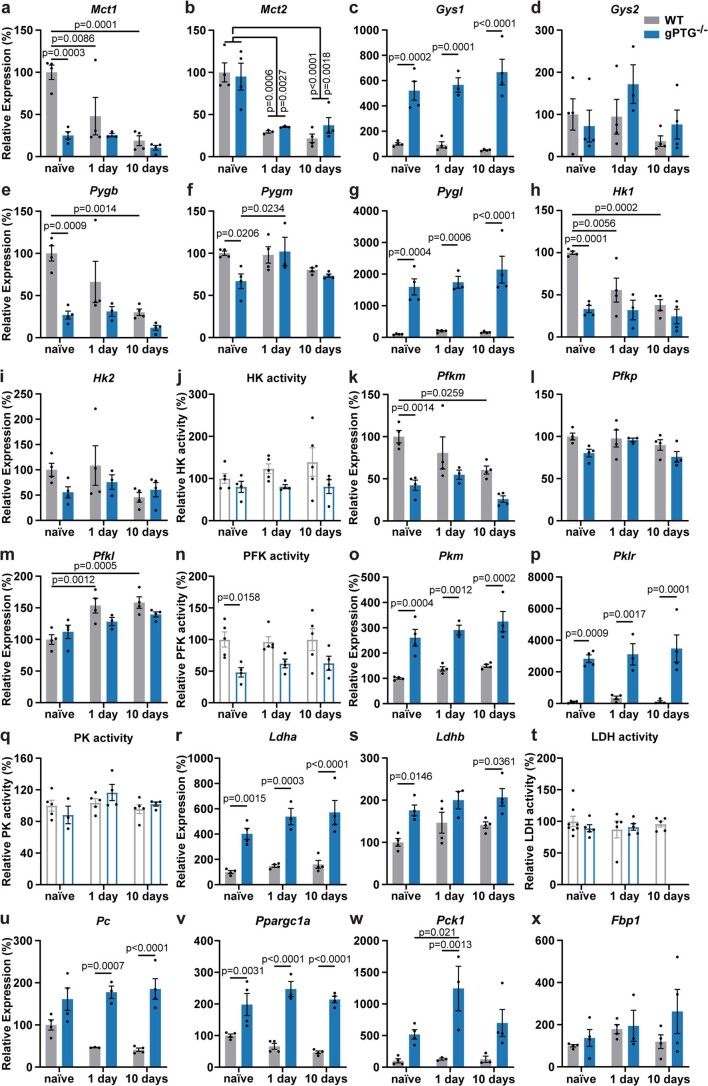
Expression of genes related to glucose and lactate metabolism are modulated by an inflammatory pain stimulus and altered in gPTG^−/−^ mice. **(a, b)** qPCR expression analysis of monocarboxylate transporters 1 and 2 in dorsal spinal cord tissue of WT and gPTG^−/−^ mice before and at 1 and 10 days after CFA stimulation, relative to WT naïve mice. N = 4. **(c-g)** qPCR expression analysis of glycogen metabolism related genes in dorsal spinal cord tissue of WT and gPTG^−/−^ mice before and at 1 and 10 days after CFA stimulation, relative to WT naïve mice. N = 4. **(h, i)** qPCR expression analysis of hexokinase genes in dorsal spinal cord tissue of WT and gPTG^−/−^ mice before and at 1 and 10 days after CFA stimulation, relative to WT naïve mice. N = 4. **(j)** Hexokinase enzymatic activity in dorsal spinal cord tissue of WT and gPTG^−/−^ mice before and at 1 and 10 days after CFA stimulation, normalized to WT naïve mice. N = 5. **(k-m)** qPCR expression analysis of phosphofructokinase genes in dorsal spinal cord tissue of WT and gPTG^−/−^ mice before and at 1 and 10 days after CFA stimulation, relative to WT naïve mice. N = 4. **(n)** Phosphofructokinase enzymatic activity in dorsal spinal cord tissue of WT and gPTG^−/−^ mice before and at 1 and 10 days after CFA stimulation, normalized to WT naïve mice. N = 5. **(o, p)** qPCR expression analysis of pyruvate kinase genes in dorsal spinal cord tissue of WT and gPTG^−/−^ mice before and at 1 and 10 days after CFA stimulation, relative to WT naïve mice. N = 4. **(q)** Pyruvate kinase enzymatic activity in dorsal spinal cord tissue of WT and gPTG^−/−^ mice before and at 1 and 10 days after CFA stimulation, normalized to WT naïve mice. N = 5. **(r, s)** qPCR expression analysis of lactate dehydrogenase (Ldh) genes in dorsal spinal cord tissue of WT and gPTG^−/−^ mice before and at 1 and 10 days after CFA stimulation, relative to WT naïve mice. N = 4. **(t)** LDH enzymatic activity in dorsal spinal cord tissue of WT and gPTG^−/−^ mice before and at 1 and 10 days after CFA stimulation, normalized to WT naïve mice. N = 5. (**u-x**) qPCR expression analysis of gluconeogenesis related genes in dorsal spinal cord tissue of WT and gPTG^−/−^ mice before and at 1 and 10 days after CFA stimulation, relative to WT naïve mice. N = 4. Two-way ANOVA with Bonferroni post hoc test. Data represent mean ± s.e.m.

Interestingly, among the genes most robustly transcriptionally altered subsequent to an inflammatory pain stimulus were the monocarboxylate (lactate) transporters *Mct1* and *Mct2*. Expression levels of both transporters were reduced in spinal tissue of WT mice subsequent to CFA treatment. Intriguingly, astrocytic *Mct1* was significantly lower expressed and not modulated by the inflammatory pain stimulus in gPTG^−/−^ mice (Extended Data Fig. 7a,b). Although alterations of lactate levels alone are unlikely to explain all of the observed phenotypes, these results provide further evidence that pain-induced spinal activation modulates lactate metabolism and transport and that astrocytic PTG-dependent glycogen metabolism is a relevant contributor to this process.

**Extended Data Fig. 8:**
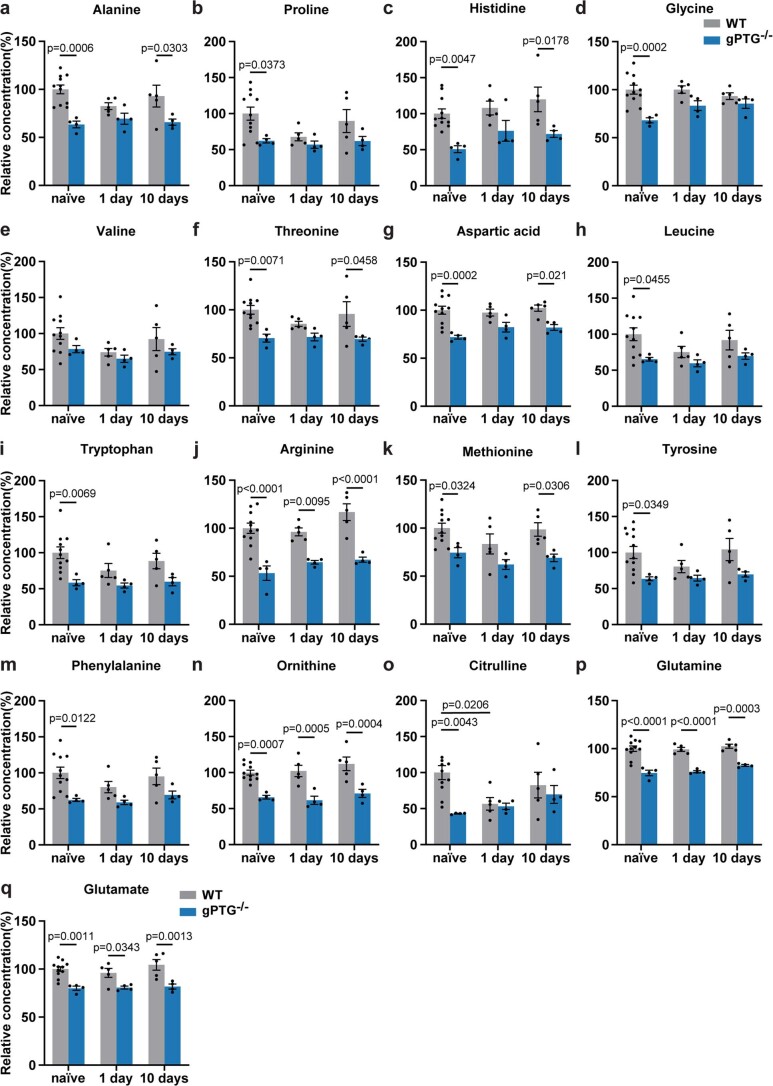
Amino acid content is modulated by CFA injection and is overall decreased in gPTG^−/−^ in the dorsal spinal cord. **(a-q)** Amino acid content in dorsal spinal cord tissue of WT and gPTG^−/−^ mice before and at 1 and 10 days after CFA stimulation, relative to WT naïve mice. N = 4 samples of 4 mice. Two-way ANOVA with Bonferroni post hoc test. Data represent mean ± s.e.m.

When we focused our attention on the transcriptional analysis and activities of major (rate-limiting) enzymes involved in glycogen and glucose metabolism in spinal tissue, we found that several of these are altered in gPTG^−/−^ mice (Extended Data Fig. 7). While expression of the glycolytic enzyme phosphofructokinase (*Pfkm*) and activity of PFK were reduced in gPTG^−/−^ mice, pyruvate kinases and lactate dehydrogenases were transcriptionally upregulated in gPTG^−/−^ mice as compared with WT controls. However, the changes in relative expression were not recapitulated in pyruvate kinase and lactate dehydrogenase activity changes (Extended Data Fig. 7k–t). These results could indicate that alternative metabolic pathways are engaged to compensate for reduced glucose metabolites in gPTG^−/−^ mice. This is in agreement with three of the four rate-limiting gluconeogenic enzymes (PyrCar, PGC1alpha and PEPCK-1) being upregulated in gPTG^−/−^ mice as compared with WT controls (Extended Data Fig. 7u–x). Additionally, a reduced abundance of several glucogenic amino acids in spinal tissue of gPTG^−/−^ mice as compared with WT controls is also concurrent with this hypothesis (Extended Data Fig. 8). Intriguingly, among the amino acids displaying lowered levels is glutamate, the main excitatory transmitter that has been implicated in spinal plasticity and the development of chronic forms of pain^53^.

In summary, these data suggest that interconnected, glucose-related catabolic and anabolic pathways modulate maladaptive plasticity in the spinal nociceptive pathway to shape long-lasting inflammatory pain in a PTG- and glycogen-dependent manner.

**Extended Data Fig. 9:**
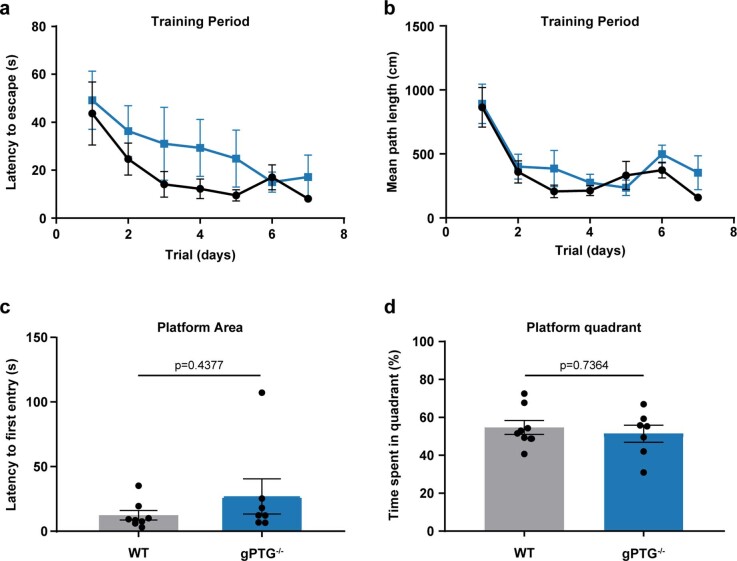
Basic learning and memory is similar in wildtype and PTG^−/−^ mice in the Morris Water Maze (MWM) test. **(a**, **b)** Latency to escape from swimming in the water and to reach a platform **(a)** and average distance traveled until the platform was reached **(b)** during the training period in the Morris Water Maze (MWM) of wildtype (WT) and gPTG^−/−^ mice. N = 8 mice per group; Two-way ANOVA with Bonferroni post hoc test. **(c, d)** Latency to first entry the platform area **(c)** and percent of time WT and gPTG^−/−^ spent in the quadrant of the platform **(d)** during the last test of the MWM (8 days after start of training) when the platform has been removed. No differences in learning and memory where observed. Unpaired two-tailed t-test; N = 8 mice per group; Data represent mean ± s.e.m.

Since beneficial forms of plasticity in higher brain centres—associated with learning and memory—have also been shown to depend on astrocytic metabolism, we wondered whether PTG^−/−^ mice are compromised in cognitive memory formation. We therefore assessed the ability of the mice to perform in the Morris water maze test, a classic paradigm to assess spatial learning and memory in rodents^54^. We found that PTG^−/−^ mice performed similarly to WT control mice in this assay (Extended Data Fig. 9), suggesting that the metabolic PTG pathway does not majorly contribute to this learning and memory paradigm.

## Discussion

Brain astrocytes have been suggested to provide metabolites and metabolic energy for neighbouring neurons in situations when additional energy is required, such as forms of long-term plasticity and memory formation^32–39^.

Plasticity of spinal neurons, such as long-term potentiation and enhanced excitability of L1 neurons of the pain pathway, contributes to persistent forms of pain^43,45,47,55^. While the induction and acute phase of spinal plasticity has been researched extensively and several molecular mechanisms have been identified, mechanisms prolonging this state of maladaptive plasticity and thus enabling transition to persistent pain are yet to be fully understood.

Astrocytes in the spinal cord have been shown to mediate long-term nociceptive sensitization in models of inflammatory pain^6,8^. Together with microglia, astrocytes have also been described to contribute to pain-induced, glia-mediated forms of spinal plasticity^56^. Importantly, while glial contributions have been identified in terms of molecular signalling and release of pro-inflammatory mediators, they have not been tested in terms of driving metabolic plasticity relevant to pain.

We show here that spinal astrocytes, triggered by painful stimuli, adjust their energy metabolism to sustain and maintain long-lasting inflammatory pain states.

Previous work has provided evidence that metabolic inhibitors blocking the tricarboxylic acid cycle, such as fluorocitrate and fluoroacetate, are effective at inhibiting pain in rodent models. Intriguingly, these metabolic blockers are preferentially taken up by astrocytes, suggesting that the metabolic perturbation of astrocytes can have beneficial effects and alleviate persistent forms of pain^6,57^. However, these drugs are only partially selective and, eventually, result in astrocyte decline, complicating the interpretation of these results.

Our study uncovers a genetically defined pathway that, unexpectedly, drives glycogen dynamics in spinal astrocytes subsequent to inflammatory pain stimulation. Noxious stimulation-induced acute glycogen mobilization, subsequent PTG induction and glycogen build-up follow a long-term trajectory that scales with the magnitude/severity and duration of the inflammatory pain stimulus and that, according to our data, is less relevant for acute pain-related behaviours in mice but more important for establishing and/or maintaining long-term inflammatory pain. We show that blunting PTG-driven spinal glycogen accumulation reduces nociceptive neuronal plasticity in the spinal cord and, presumably as its consequence, shortens the maintenance phase of inflammatory pain states, as assessed in behavioural experiments. Our data do not rule out the possibility that baseline (non-pain-induced) PTG and glycogen levels in spinal neurons and/or other types of glia also contribute to spinal metabolism and noxious signal processing.

Our energy metabolic measurements and electrophysiological recordings conjointly emphasize robust neuronal–astrocyte metabolic interaction, whereby pain-induced neuronal activity (directly or indirectly) triggers astrocytic glycogen dynamics, thereby promoting increased glycolytic capacity to reinforce pain maintenance.

We can only speculate about the nature of such a signal that triggers the metabolic changes in astrocytes. It is possible that dorsal spinal astrocytes respond directly to neurotransmitters released by primary afferent fibres, such as glutamate and/or inflammatory mediators^6^. Interestingly, it has been shown that afferent pain signals acutely activate spinal astrocytes indirectly via the release of noradrenaline from descending fibres originating in the locus coeruleus^8^. Noradrenaline, but also insulin, glutamate and other soluble signalling molecules, can stimulate an increase in glycogen content in cultured astrocytes^58–63^, a property that has been linked to PTG induction in vitro^23^. It is therefore conceivable that neuronal activity of primary afferent nociceptive neurons promotes astrocytic glycogen build-up by a soluble signalling molecule released in the spinal cord.

But how can astrocytic glycogen build-up and accumulation result in a protracted inflammatory pain state? A potential explanation is offered by a recent study that analysed PTG^−/−^ adipocytes^64^. Similar to astrocytes, stimulation of adipocytes with noradrenaline can also promote PTG-dependent glycogen build-up. However, it was found that not the build-up per se, but rather the build-up coupled with glycogen turnover—glycogen dynamics—is essential for cellular function, a concept which has previously been hinted at by studies that had detected glycogen ‘over-accumulation’ in adipose tissue^65,66^. We hypothesize that a similar phenomenon also prevails in spinal astrocytes in the course of pain signal processing, in that it is increased glycogen dynamics, rather than solely glycogen accumulation, which is relevant for the cellular and behavioural phenotypes we observe. This probably explains why we find glycolytic capacity to be reduced in PTG^−/−^ spinal cord tissue: the lack of noxious stimulation-induced glycogen build-up in the absence of PTG results in reduced substrate availability for glycolysis (glucose generated via glycogenolysis) and thereby prevents an increase in glycolytic capacity as compared with the WT dorsal spinal cord network.

In such a scenario, spinal glycogen dynamics would act as a metabolic buffer and noxious stimuli-boosted glycolytic capacity in the spinal cord of WT mice would provide metabolites that may signal and/or calorically fuel spinal plasticity.

Among the different metabolites that are exchanged between astrocytes and neurons, lactate has received considerable attention. Since the 1990s it has been postulated that astrocytic glycogen may constitute a source for lactate (rather than for glucose) for neighbouring neurons^33^. Lactate can modulate excitability of some neurons via its caloric value^46,67^ and also in its capacity as an extracellular signalling molecule^51,68,69^. Additionally, the metabolite was found to foster memory formation in higher brain centres^39^, a result that promoted the so-called astrocyte–neuron lactate-shuttle hypothesis (ANLSH), whereby lactate transfer fuels neuronal plasticity.

Our electrophysiological data point to a reduction of neuronal plasticity and diminished noxious stimulation-induced excitability in the absence of astrocytic glycogen dynamics. Although our metabolomics analysis suggests that spinal lactate levels are altered during pain stimulation and are reduced in PTG^−/−^ mice, we have no evidence that ANLSH is relevant in driving this type of spinal plasticity. While blocking monocarboxylate (lactate and pyruvate) transporters reduced inflammatory pain-induced hyperexcitability in spinal L1 neurons, lactate supplementation to gPTG^−/−^ spinal slices obtained from mice stimulated with CFA was not sufficient to promote hyperexcitability.

Although it is possible that application of supraphysiological lactate to spinal slices inhibits neuronal excitability by engaging lactate (HCA) receptors^51,68,69^, pyruvate also failed to induce hyperexcitability in our electrophysiological experiments^46^, suggesting that hyperexcitability cannot be rescued solely by acutely increasing extracellular levels of these metabolites with caloric value. Collectively, these results suggest that ANLSH does not play a major role in mediating spinal hyperexcitability, at least not after inflammatory pain-induced spinal plasticity has already been established.

It is possible that glycogen dynamics and lactate shuttling may be relevant early on during the inflammatory insult, to prime and fuel structural plasticity in the spinal cord that eventually results in hyperexcitability (and enhanced pain maintenance) at later stages. Manipulating lactate levels (and lactate transfer) after the plastic changes have been established—as we did in our slice electrophysiology experiments—would then not have any major effect.

Our results showing that perfusion of MCT inhibitors can inhibit CFA-induced hyperexcitability in spinal slices can also be explained without invoking the ANLSH model and may indicate an inhibitory role of endogenous lactate: MCT inhibition may reduce lactate uptake into cells, thereby increasing extracellular lactate levels, which could reduce neuronal activity via HCA receptor signalling^51,68,69^.

It is also possible that lactate plays only a minor role in this process. Glycogen metabolism and glycolysis provide metabolites for many metabolic pathways and ‘building blocks’ for the generation of signalling molecules. It is therefore likely that the altered abundance of other metabolites besides lactate contributes to reduce spinal plasticity in PTG^−/−^ mice as compared with WT control animals. We found several amino acids to be reduced in PTG KO animals, including the excitatory transmitter glutamate and its precursor glutamine (Extended Data Fig. 8). Different to ANLSH, the glutamate–glutamine cycle is a well-documented metabolic interaction between astrocytes and neurons^70^. One intriguing possibility is that the ratio between glutamate and the main inhibitory transmitter gamma aminobutyric acid (GABA), both of which have been implicated in the establishment of persistent forms of pain^53,71^, is altered in PTG^−/−^ mice, thereby affecting pain-induced spinal plasticity and hyperexcitability.

The complexity of the spinal neuroglia network and the current lack of suitable (in vitro) models recapitulating the cellular interactions and compartmentalization of the spinal cord limited the metabolic analysis of our study. Future work, requiring cell-type-selective metabolic flux measurements and refined metabolic ex vivo and in vivo perturbations, combined with neuroglia activity and plasticity measurements, will help to shed light onto this clinically highly relevant phenomenon, which may extend to other CNS circuits involved in pain signal processing.

Interestingly, PP1, the signalling molecule regulated by PTG to control glycogen content, has previously been implicated in neuronal plasticity in higher brain centres to foster learning and memory^72–75^. However, we did not find any gross abnormalities in the PTG-deficient animals, suggesting that other metabolic mechanisms are at play to fuel these types of plasticity. Given that we find glycogen dynamics in spinal astrocytes to parallel the magnitude of the pain stimulus, we speculate that more subtle sensory stimuli that underlie learning and memory in higher brain centres—and following different kinetics compared with long-lasting noxious stimuli—probably correlate with more subtle changes in the energetic landscape that are independent (or less dependent) on the transcriptional induction of PTG.

Future research will tell whether the spinal astrocyte–neuron metabolic interaction that we discovered here will constitute a viable therapeutic target to prevent (or revert) maladaptive neuronal plasticity associated with the maintenance of long-term pain states.

## Methods

### Mice

All animal care and experimental procedures were approved by the local council (Regierungspräsidium Karlsruhe, Germany) under protocol numbers 35-9185.81/G-168/15, 35-9185.81/G-201/16, 35-9185.81/G-295/21, 35-9185.81/G-173/21. Mice were housed with food and water ad libitum under a standard 12-h light/dark cycle (light on between 07:00 and 19:00) with regulated ambient temperature of ±22 °C and at relative humidity of 35–45%. All genetically modified mice in this study were on the C57BL/6N background. All mice were bred at the animal facility of Heidelberg University (Interfakultäre Biomedizinische Forschungseinrichtung, IBF) or purchased from Janvier Labs. Both male and female mice were used for the experiments. The animals were randomly assigned for experiments and the experimenter was aware of the animal genotype when conducting experiments, except for behaviour tests. Mice aged 7–16 weeks were used, except for Seahorse experiments where mice were 3–5 weeks old. Before experiments, mice were given 24–72 h to habituate in the laboratory.

### Mouse lines used

All WT animals used in this study correspond to the C57BL/6N mouse line.

Astrocyte-specific conditional (cPTG^−/−^) and global (gPTG^−/−^) KO mouse lines were made using standard transgenic methods as described by Irimia et al.^76^ (see Extended Data Fig. 3a for details). In brief, embryonic stem cells were initially incubated with G418 for positive selection via the neo gene and utilizing the diphtheria toxin gene as negative selection marker. Surviving cell clones were PCR-screened for targeted integration and positive clones further verified by Southern blot analysis to ensure that they contained the three LoxP sites and that the *Ptg* locus was accurately targeted. A phosphoglycerate kinase-nuclear localization signal (NLS)/Cre plasmid was electroporated into two separate clones; subsequently, the cells were cultured in medium containing ganciclovir, which eliminated cells that still contained thymidine kinase. As a result, only the cells that were either complete KO or conditional *Ptg* KO survived, the latter of which having the marker genes removed but retaining two loxP sites surrounding the floxed gene region (refer to Extended Data Fig. 3a for details). PCR analysis revealed appropriate conditional KO cells. Post-karyotyping, two distinct conditional embryonic stem cell clones were used to generate two independent mouse lines by injection into C57Bl/6J blastocysts. The male chimeric mice, identifiable by their agouti coat colour, were bred with C57Bl/6j mice and germline transmission confirmed by genotyping PCR. Frozen sperm from these mice were used for in vitro fertilization, to regenerate PTG^+/f^ mice, which were backcrossed to C57Bl/6N mice.

PTG^+/fl^ mice were crossed with a deleter-Cre strain provided by Reinhard Fässler from Max-Planck-Institut für Biochemie^28^ to eliminate the loxP flanked region, generating PTG^+/−^ animals. Global (general) gPTG^−/−^ mice where then generated by inbreeding of PTG^+/−^.

Astrocyte-specific conditional PTG KO animals, cPTG^−/−^, were obtained by crossing two lines: first, a homozygous floxed PTG line (PTG^fl/fl^) was generated by inbreeding of PTG^+/fl^, giving PTG^fl/fl^ mice as a result. Second, gPTG^−/−^ mice were crossed with an astrocyte-specific Tamoxifen-dependent line (Aldh1L1-Cre^ERT2^) obtained from the laboratory of Bal Khakh at the University of California, Los Angeles^29^, generating Aldh1L1-Cre^ERT2^-PTG^+/−^ mice. Finally, this line was crossed with PTG^fl/fl^, producing Aldh1L1-Cre^ERT2^-PTG^fl/−^ mice.

cPTG^−/−^ mice were finally generated by intraperitoneally injecting 200 µl of a solution containing 10 mg ml^−^^1^ tamoxifen (Sigma, T56485) dissolved in a mixture of 5% ethanol and 95% sunflower oil (Sigma, 88921). Animals were injected once a day for 5 consecutive days. Littermate PTG^+/fl^ mice that did not express Aldh1L1-Cre^ERT2^ were injected with tamoxifen at the same time and used as a control group for our experiments. Experiments were conducted only 3 weeks after the last injection.

### Pain models

#### FA pain model

The 36.5% formaldehyde solution (Sigma, F8775) was diluted with 0.9% NaCl sterile saline (B. Braun, 190/150936) to a 5% solution shortly before injection. FA-induced pain was induced by intraplantar injection of 15 µl of the 5% formaldehyde solution into the hindpaw of the mouse, under shallow isoflurane anaesthesia.

#### Capsaicin pain model

The 0.6% capsaicin/dimethylsulfoxide solution was diluted with sterile saline to a 0.06% suspension shortly before injection. Capsaicin-induced pain was induced by intraplantar injection of 20 µl of the 0.06% capsaicin solution into the hindpaw of the mouse, under shallow isoflurane anaesthesia.

#### Serotonin pain model

A 0.5% serotonin/0.9% NaCl solution was used. Serotonin-induced pain was induced by intraplantar injection of 20 µl of the serotonin solution into the hindpaw of the mouse, under shallow isoflurane anaesthesia.

#### PGE2 pain model

A 2.5 ng µl^−^^1^ PGE2/0.9% NaCl solution was used. PGE2-induced pain was released by intraplantar injection of 20 µl of the PGE2 solution into the hindpaw of the mouse, under shallow isoflurane anaesthesia.

#### CFA pain model

CFA (20 µl) (Sigma, F5881) was injected into the hindpaw while the mouse was kept under shallow anaesthesia by isoflurane, to induce inflammatory pain.

#### SNI pain model

The SNI surgery was performed by severing two of the three branches of the sciatic nerve (the tibial nerve and the common peroneal nerve). The mice were kept under isoflurane anaesthesia during surgery and allowed to recover for at least 1 week before being subjected to any measurements/experiments.

### Wholemount immunofluorescence staining

#### Sample preparation

Mice were killed 2 h after intraplantar FA injection and immediately transcardially perfused with PBS followed by 4% paraformaldehyde (PFA). After perfusion, the spinal cord was dissected out and subjected to 4 h of postfixation in 4% PFA/PBS at 4 °C while gently rotating. The tissue piece was then washed in PBS for 10 min and put into Dent’s Bleach (10% H_2_O_2_ (Merck, 107209), 13.3% dimethylsulfoxide (Sigma, D2438) and 53.3% methanol (Honeywell, 32213)) for 24 h at 4 °C while gently rotating. After bleaching, the tissue piece was dehydrated using methanol, replacing it five times after 2 min each time. The sample was then fixed in Dent’s Fix (20% dimethylsulfoxide, 80% methanol) for 24 h at 4 °C with gentle rotation and then stored in Dent’s Fix at 4 °C until further use.

#### Staining procedure

The processed tissue was rinsed three times with PBS (20 min each) to wash off the Dent’s Fix and then incubated with primary antibody in blocking buffer (pS6, Cell Signaling 2215, 1:1,000) at 4 °C with gentle rotation for 5 d. After primary antibody incubation, the tissue sample was washed six times with PBS (30 min each time) at room temperature. Secondary antibody (diluted in blocking solution) Donkey αRabbit (Invitrogen, A21206, 1:2,000) was added and incubated for 2 d at room temperature while gentle rotating. After the incubation, the tissue was washed six times with PBS (30 min each time) at room temperature.

#### Clearing procedure

After staining, the spinal cord piece was dehydrated and cleared before imaging. Dehydration was done by incubating the tissue in 50% methanol/PBS for 5 min at room temperature, followed by 20 min of incubation in 100% methanol, changing the solution twice during the incubation. After dehydration, the tissue was cleared in BABB (1 part benzyl alcohol (Sigma, 108006) and 2 parts benzyl benzoate (Sigma, B6630)) for 5 min and stored in BABB until imaging.

### Immunoprecipitation/ribosome capture

#### Sample preparation

For sequencing experiments, five mice were pooled for each sample. Three samples of each condition were prepared. Immunoprecipitation was performed based on the protocol developed by Knight et al.^11^. In brief, mice were killed 2 h after FA injection and spinal cords quickly dissected into freshly prepared, ice-cold buffer B (1 × HBSS, Life Technologies, 14185-045), 4 mM NaHCO_3_ (AppliChem, 131638.1211), 2.5 mM HEPES (pH 7.4; Roth, 9105.4), 35 mM glucose (Merck, 08337), 100 µg ml^−^^1^ cycloheximide (Sigma, C7698). The tissue was snap-frozen and kept at −80 °C until homogenization.

#### Homogenization

Tissue was resuspended in 1,350 µl of freshly prepared buffer C (10 mM HEPES (pH 7.4), 150 mM KCl (Sigma, P9541), 5 mM MgCl_2_ (Sigma, M2670), 1 × PhosSTOP phosphatase inhibitor tablet (Sigma, 4906845001), 1 × cOmplete Protease Inhibitor Cocktail (Roche, 11697498001), 2 mM dithiothreitol (Sigma, 10197777001), 100 U ml^−1^ RNasin (Promega, N2515), 100 µg ml^−^^1^ cycloheximide)) and homogenized at 4 °C. The homogenates were centrifuged at 2,000*g* for 10 min at 4 °C and the supernatants (~1 ml) were transferred to fresh tubes on ice. Then, 90 µl of 10% NP40 and 90 µl of freshly prepared 1,2-diheptanoyl-sn-glycero-3-phosphocholine (DHPC; Avanti Polar Lipids, 850306, 100 mg/0.69 ml) were added to each sample. The mixture was centrifuged at 17,000*g* for 10 min at 4 °C and the supernatant was transferred to a fresh tube. A small aliquot of the supernatant was taken for western blot (‘Ly’) and the rest was used for immunoprecipitation.

#### Antibody coating

For each immunoprecipitation, 3 µg of pS6 (Cell Signaling, clone 5364) antibody and 50 µl of Protein A Dynabeads (Invitrogen, 1001D) were used. Antibody was incubated with Dynabeads in 1 ml of buffer A (0.1% Triton-X-100/PBS) for 10 min at room temperature with gentle rotation. The coated beads were equilibrated in buffer C (see above) until immunoprecipitation.

#### Immunoprecipitation

Immunoprecipitation was performed by applying the supernatant to pS6-coated protein A Dynabeads and rotating at 4 °C for 10 min. After immunoprecipitation, the liquid was collected for western blot (‘SN’). The beads were washed four times in 500 µl of buffer D (10 mM HEPES (pH 7.4), 350 mM KCl, 5 mM MgCl_2_, 2 mM dithiothreitol, 1% NP40, 100 U ml^−^^1^ RNasin and 100 µg ml^−^^1^ cycloheximide). During the third wash, the beads were transferred to a fresh tube and incubated at room temperature for 10 min. Phospho-ribosomes were eluted with 350 µl of RLT Buffer (Qiagen, 79216), and the eluates were immediately processed to RNA extraction for test qPCR or deep sequencing experiments. Alternatively to elution, for ‘immunoprecipitate’ western blot, 50 µl of 2 × Laemmli was added to the phospho-ribosomes.

### RNA extraction

RNA extraction was performed using the RNAeasy micro kit (QIAGEN, 74004) following the manufacturer’s instructions. The elution volume was 10 µl. The RNA was reverse-transcribed using SuperScript III Reverse Transcriptase (Thermo Fisher, 18080044) and Oligod(T)23VN (NEB, S1327) following the manufacturer’s instructions for the SuperScript First-Strand Synthesis System (Thermo Fisher).

### RNA-seq

RNA was extracted from immunoprecipitate eluates as described above. The obtained RNA was snap-frozen and kept at −80 °C until further use.

#### Reverse transcription and amplification

Complementary DNA synthesis and amplification were performed following the SMART-Seq2 protocol described in ref. ^77^ and optimized by EMBL Genecore. We used 2 µl of RNA as starting material and 18 cycles of amplification were performed.

#### Quality control

The amplified cDNA was subjected to concentration measurement using the Qubit dsDNA HS Assay kit (Thermo Fisher, Q32854) and a quality check using a bioanalyzer (Agilent High Sensitivity DNA Kit, 5067-4626). A small aliquot of the amplified cDNA was used for qPCR testing while the remaining cDNA was kept at −20 °C until further processing.

#### Library preparation, barcoding and sequencing

Library preparation and barcoding were performed following the standard protocol of EMBL Genecore (Nextera XT DNA Library Preparation Kit, FC-131-1096, and Nextera XT Index Kit v2 Set A, FC-131-2001), using 125 pg of amplified cDNA as input. The sequencing was performed by EMBL Genecore on an Illumina MiSeq platform.

### qPCR

qPCRs were performed using the FastStart Essential DNA Green Master (Roche, 06402712001) on a Roche LightCycler 96 Instrument, following the manufacturer’s instructions.

#### qPCR primers used

*Ptg (Ppp1r3c)*-Fw: GGTGACTCATCTTTCTGCCACA

*Ptg (Ppp1r3c)*-Rv: CAAGACAAAATTAGGCACGAGA

*Tubb3*-Fw: TGAGGCCTCCTCTCACAAGT

*Tubb3*-Rv: GTCGGGCCTGAATAGGTGTC

*Hk1*-Fw: CACAAGACACCCACAGGGAT

*Hk1*-Rv: GCATACGTGCTGGACCGATA

*Hk2*-Fw: CAAGTGCAGAAGGTTGACCA

*Hk2*-Rv: GTGTGTGGTAGCTCCTAGCC

*Mct1*-Fw: AGTGCAACGACCAGTGAAGT

*Mct1*-Rv: AGATACTGCTGATAGGACCTCCA

*Mct2*-Fw: ATGTATGCGGGAGGTCCCAT

*Mct2*-Rv: GGCTGGTTGCAGGTTGAATG

*Ldha*-Fw: GTACACGGAGACCTCGGTATTAT

*Ldha*-Rv: CATCCGCCAAGTCCTTCATT

*Pfkp*-Fw: ACTATCACAGACACGTGCGA

*Pfkp*-Rv: GCCAGGTAGCCACAGTATCC

*Pfkm*-Fw: CACAAGACACCCACAGGGAT

*Pfkm*-Rv: GCATACGTGCTGGACCGATA

*Pfkl*-Fw: CGCTGCAATGGAGAGTTGTG

*Pfkl*-Rv: CGCTGCAATGGAGAGTTGTG

*Pkm*-Fw: ATGCAGCACCTGATTGCCC

*Pkm*-Rv: CGGCGGAGTTCCTCGAATAG

*Pklr*-Fw: GCAACATGCGATTGCCCG

*Pklr*-Rv: ATTGCAGTGACCTCGGTTGG

*Gys1*-Fw: ATCTACACTGTGCTGCAGACG

*Gys1*-Rv: CCCTTGCTGTTCATGGAATCC

*Gys2*-Fw: CCATCCTCAGCACCATTAGAC

*Gys2*-Rv: GTGACAACCTCGGACAAACTC

*Pygm*-Fw: ATCAACCCCAACTCGCTCTTT

*Pygm*-Rv: GCTCCCTTTTGATGCGGTT

*Pygb*-Fw: CAGCAGCATTACTATGAGCGG

*Pygb*-Rv: CCAAGTCCAACCCCAACTGA

*Pygl*-Fw: TGCTTTGGATAAGAAGGGGTATGAGGC

*Pygl*-Rv: TTGAAGAGGTCTGGCTGATTGGGAG

*Ldhb*-Fw: AAAGGCTACACCAACTGGGC

*Ldhb*-Rv: GCCGTACATTCCCTTCACCA

*Pc*-Fw: TGCCAAGCAGGTAGGCTATGA

*Pc*-Rv: GCGGGAATTGACCTCGATGAA

*Pck1*-Fw: GGCGATGACATTGCCTGGATGA

*Pck1*-Rv: TGTCTTCACTGAGGTGCCAGGA

*Ppargc1a*-Fw: GAATCAAGCCACTACAGACACCG

*Ppargc1a*-Rv: CATCCCTCTTGAGCCTTTCGTG

*Fbp1*-Fw: TGCTGAAGTCGTCCTACGCTAC

*Fbp1*-Rv: TTCCGATGGACACAAGGCAGTC

### Immunofluorescence staining

#### Sample preparation

Mice were perfused first with PBS (Sigma, D8537) then 4% paraformaldehyde/PBS (Sigma, 16005). After perfusion, the mice were dissected to collect the lumbar spinal cord and this was embedded in OCT (Tissue-Tek O.C.T. Compound, Sakura, 4583). The sample was sectioned to 20 µm on a Leica CM3050S Research Cryostat and collected on glass slides (HistoBond, 0810001). The sections were stored at −80 °C until further use.

#### Staining

The sections were first allowed to warm to room temperature and then washed in PBS-X (0.2% Triton-X (Merck, 108603)/PBS) twice for 5 min each time. Blocking was performed with 1% horse serum in PBS-X for 1 h at room temperature. The sections were incubated with primary antibody in blocking buffer overnight at 4 °C. After washing in PBS-X four times, for 15 min each, secondary antibodies (1:1,000 in blocking buffer) and DAPI (1:100) were applied to the sections and incubated at room temperature for 2 h. The sections were mounted using Immu-Mount (Thermo Scientific, 9990402) after another washing in PBS-X (four times, 15 min each) and preserved at 4 °C.

#### Primary antibodies

Primary antibodies used were: pS6 (Cell Signaling, 2215, 1:1,000); GFAP (Cell Signaling, 3670, 1:500); NeuN (Cell Signaling, D4G40, 1:2,000); IBA1 (Wako, 019-19741, 1:500); GFAP (Synaptic Systems, 173004, 1:1,000); GFP (Rockland, 600-101-215, 1:1,000); mCherry (Sicgen, AB0040-500, 1:5,000); Sox9 (Abcam, ab185966, 1:1,500).

#### Secondary antibodies

Secondary antibodies used were: Donkey αMouse, Alexa Fluor 594 (Dianova, 711585150); Donkey αRabbit, Alexa Fluor 488 (Invitrogen, A21206); Goat αMouse, Alexa Fluor 488 (Invitrogen, A11001); Donkey αGoat, Alexa Fluor 488 (Invitrogen, A11055); Donkey αGoat, Alexa Fluor 555 (Invitrogen, A21432).

### In situ hybridization

#### Sample preparation

Spinal cords were dissected from naive animals or from animals 2 h after stimulus (FA, capsaicin or CFA) injection into ice-cold PBS and immediately embedded in OCT and frozen using dry ice/isopropanol. Then, 20-µm sections were prepared using a Leica CM3050S cryostat, dried at 37 °C and stored at −80 °C until further use.

#### Probe design and synthesis

In situ hybridization probes were designed using the commercial software OLIGO (v.7).

#### Primers used

*Ptg*-Fw: GACGTCGACAAGAACTTTGTCTGCCTCGAGA

*Ptg*-Rv: GACGCGGCCGCTACCACAGCGTTCCATCACC

Probe sequences were amplified from spinal cord cDNA, molecularly cloned into pBlueScript plasmids and bacterially amplified. DIG-labelled in situ probes were subsequently synthesized. In situ hybridization was performed using standard procedures^78^. In brief, DIG- and/or FITC-labelled cRNA probes were used for hybridization on cryosections. Hybridization was performed overnight at 65 °C. Sections were washed at 60 °C twice in 2 × SSC/50% formamide/0.1% *N*-lauroylsarcosine, treated with 20 µg ml^−^^1^ RNAse A for 15 min at 37 °C, washed twice in 2 × SSC/0.1% *N*-lauroylsarcosine at 37 °C for 20 min and then washed twice in 0.2 × SSC/0.1% *N*-lauroylsarcosine at 37 °C for 20 min. Sections were blocked in MABT/10% goat serum/1% Blocking reagent (Roche, 11096176001). For NBT/BCIP staining, sections were incubated overnight with sheep anti-DIG-AP (1:1,000, Roche 11093274910). After washing, staining was performed using NBT/BCIP in NTMT until reaching satisfactory intensity. Double fluorescence in situ staining procedures were performed by two consecutive rounds of TSA amplification with intermediate peroxidase inactivation. Sections were incubated with sheep anti-FITC-POD (1:2,000, Roche, 18 11426346910) or sheep anti-DIG-POD (1:1,000, Roche, 11207733910). Subsequently, sections were incubated with Streptavidin-Cy2 and DAPI in blocking solution, washed and mounted in Immu-Mount (Shandon).

#### Immunofluorescence in situ hybridization double staining

Single-colour fluorescence in situ hybridization was performed following the same protocol as above. After single-colour fluorescence in situ hybridization was completed, the sections were subjected to antibody immunofluorescent staining (pS6, Cell Signaling, 2215, 1:1,000; GFAP, Cell Signaling, 3670, 1:500; NeuN, Cell Signaling, D4G40, 1:2,000; IBA1, Wako, 019-19741, 1:500) using the protocol cited above.

### RNAscope

Sample preparation was done similarly to the immunostaining protocol, with the modification that sections were prepared with a thickness of 14 µm. RNAscope v2 in situ hybridization assay was performed according to the manufacturer’s instructions (Advanced Cell Diagnostics). A custom PTG probe (838311) was designed and produced by Advanced Cell Diagnostics.

#### Immunostaining after RNAscope

The RNAscope v2 protocol was followed using Protease III instead of Protease IV. After the last wash step of the protocol, samples were washed twice in 1 × PBS and the immunostaining protocol described above was followed.

#### Nissl staining

NeuroTrace 530/615 Red Fluorescent Nissl Stain (1:100, Thermo Fisher, N21482) was used to stain neurons after immunostaining, following the manufacturer’s instructions.

### *Ptg* quantification

Confocal images obtained after RNAscope for WT, cKO^−/−^ and gPTG^−/−^ were analysed using ilastik v.1.4.0 (ref. ^79^) with the Pixel Classification workflow, and the count was performed on the resulting Probability files.

### Glycogen assay

#### Sample preparation

Animals were killed under isoflurane anaesthesia; lumbar spinal cord dorsal horn was dissected and immediately snap-frozen. The samples were kept at −80 °C until further processing. For DRG dissections/preparations, at least three DRGs were collected from the lumbar (L2–L5) section of the spinal cord of naive animals or from the corresponding ipsilateral L2–L5 spinal cord section of CFA-treated animals.

For brain sections, the animal was killed under isoflurane, and the brain was quickly released from the skull and immediately frozen with dry ice. Insula, amygdala and prefrontal cortex and S1 hindlimb sections were collected following measurements from the Allen Brain Atlas and samples were snap-frozen and kept at −80 °C until further processing.

#### Glycogen measurement

Tissue was sonicated in 120 µl of ddH_2_O and incubated on a temperature shaker at 99 °C for 10 min at 350 r.p.m. to inactivate enzymes. After heat inactivation, the homogenates were centrifuged at 18,000*g* for 10 min at 4 °C. The supernatants (100–110 µl) were collected in fresh tubes. Collected supernatant (80 µl) was mixed with 34.3 µl of hydrolysis buffer from a Glycogen Colorimetric/Fluorometric Assay Kit (Biovision, K646-100) and centrifugated at room temperature for 10 min at 18,000*g*, and then 100 µl of the supernatant was divided in two wells of a 96-well plate and incubated with (sample) and without (negative control) 1 µl of hydrolysis enzyme, respectively. The remaining steps were performed following the manufacturer’s instructions. A glycogen standard curve was prepared for each experiment to counter the kit-to-kit variances. We used 10 µl of the remaining supernatants for protein measurement (Pierce BCA Protein Assay Kit, 23225) following the manufacturer’s instructions. After glycogen and protein measurements, the glycogen level of each sample was calculated by extracting the respective negative control and then normalized to the protein content.

### Lactate assay

First, 10 µl of the supernatant obtained after the first centrifugation at 18,000*g* from the Glycogen assay was combined with 90 µl of Lactate Assay Buffer (Abcam l-Lactate Assay kit, ab65331) and separated in two wells of a 96-well plate for sample and sample background control. Lactate standard and remaining steps were performed following the manufacturer’s instructions.

### Enzymatic activity assays

#### Sample preparation

Samples were collected as described for the Glycogen assay. Before sonication, 200 µl of ddH_2_O or the respective assay buffer was added to each sample, and the sample was processed following the manufacturer’s instructions.

#### Enzymatic assays

The following assays were performed following the manufacturer’s instructions: Hexokinase Activity Assay Kit (Colorimetric, Abcam, ab136957), 6-Phosphofructokinase Activity Assay Kit (Colorimetric, Abcam, ab155898), Lactate Dehydrogenase Assay Kit (Colorimetric, Abcam, ab102526) and Pyruvate Kinase Assay Kit (Abcam, ab83432).

### Metabolomic analysis

Spinal cord dorsal horn was dissected as described above. Samples were immediately snap-frozen and three samples were pooled per tube. The samples were kept at −80 °C until being shipped in dry ice and processed by Metabolon, USA. Results were obtained as the intensity of the signal of the metabolite relative to the average of the control group (naive mice).

### Amino acid analysis

#### Sample preparation

Samples were collected as described for the Glycogen assay. Before sonication, 200 µl of ice-cold ddH_2_O was added to each sample, sonicated and then centrifuged at 3,000*g* for 20 min at 4 °C. The supernatant was collected, and the precipitate discarded. Then, 10 µl of the sample was used for measuring protein content by BCA assay for normalization of the amino acid analysis data.

#### Sample measurement

For measurement of the amino acids and the acylcarnitines, a 4.7-mm disk was punched out of a blank filter card (Whatman 903 paper) in a 96-well filterplate. A total of 5 µl of tissue homogenate was given onto the disk and dried overnight at room temperature. Sample preparation was performed with the reagents of the MassChrom Kit for analysis of Amino Acids and Acylcarnitines (57000F, non-derivatized, Chromsystems) with the following steps: 150 µl of a dilution of the Internal Standards (Internal Standard – Succinylacetone:internal Standard, 1:1, v-v) and 75 µl of the Extraction Buffer – Succinylacetone were given onto the disk. The analytes were extracted by 30 min of incubation at 45 °C and 600 r.p.m. on a thermoshaker (Biosan Riga). After centrifuging at 3,200*g* for 2 min in a 96-well plate (V-bottom), the plates were incubated for 20 min at room temperature. For the measurement, 10 µl of the supernatant was injected into the tandem mass spectrometry system via flow-injection (FIA-MS/MS).

Amino acids and acylcarnitines were determined by electrospray ionization tandem mass spectrometry (ESI-MS/MS) using a Waters Xevo TQD triple quadrupole mass spectrometer equipped with an electrospray ion source and a Micromass MassLynx data system.

### Virus injections for behavioural tests

PTG^fl/fl^ mice (8–12 weeks old) were anaesthetised by intraperitoneally injecting a mixture of fentanyl (0.01 mg kg^−^^1^), medetomidine (0.3 mg kg^−^^1^) and midazolam (4 mg kg^−^^1^). After laminectomy, 500 nl of a mixture of AAV viral stocks and 20% Mannitol 2:1 v/v was injected directly into the spinal parenchyma of spinal segments L3–L4 on each side using a glass pipette, placed with an angle at 45°, and a microprocessor-controlled minipump (World Precision Instruments) at a flow rate of 100 nl min^−^^1^. Finally, the skin was sutured and the anaesthesia was antagonized with intraperitoneal injection of a mixture of naloxone (0.4 mg kg^−^^1^), flumazenil (0.5 mg kg^−^^1^) and atipamezole (2.5 mg kg^−^^1^). For postsurgery analgesia the mice received intraperitoneal carprofen (5 mg kg^−^^1^). Mice were left to recover for at least 3 weeks after surgery before use in behavioural experiments. AAV viral stocks used were ready-to-use AAV5 particles produced from pAAV.GFAP.eGFP.WPRE.hGH (105549-AAV5, Addgene) or pssAAV-2-hGFAP-mCherry_iCre-WPRE-hGHp(A) (223, VVF Zurich).

### Behavioural tests

For behavioural studies, littermate mice were used (for example, for experiments using gPTG^−/−^ mice, the control group (gPTG^+/+^/WT mice) and the experimental group (cPTG^−/−^ mice) were generated from the same cPTG^+/−^ breedings) and the experimenter was blinded to the genotype. Mice were brought into the behavioural room half an hour before beginning with the behavioural tests.

#### Mechanical stimulation of mouse hindpaws (von Frey test)

The von Frey filaments (North Coast Medical) with increasing bending forces of 0.07, 0.16, 0.4, 0.6, 1 and 1.4 g were consecutively applied to the plantar surface centre of both hindpaws. Mice were kept in standard plastic modular enclosures on top of a perforated metal platform (Ugo Basile), enabling the application of von Frey filaments from below. Mice were acclimatized on 3 consecutive days for 1.5 h to the von Frey grid and 30 min before starting the measurements on an experimental day. Each filament was tested five times on the right paw with a minimum 1-min resting interval between each application, and the number of withdrawals was recorded. Mechanical sensitivity was expressed as percentage response frequency to each filament or as 60% response threshold (g), defined as the minimum pressure required for eliciting three out of five withdrawal responses (flinching, licking or guarding the paw).

#### Heat stimulation of mouse hindpaws (Hargreaves test)

Heat sensitivity was assessed by evaluating the hindpaw withdrawal latency in response to radiant heat with the Hargreaves apparatus (Ugo Basile). Mice were kept in standard plastic modular enclosures on top of a glass platform (Ugo Basile), enabling the application of the radiant heat source (infrared intensity 40) to the hindpaw plantar surface. Mice were acclimatized on 3 consecutive days for 1 h to the setup and during at least 30 min before starting the measurements on each experimental day. Three measures were taken on each paw with a minimum 5-min resting interval between the stimulations and a cutoff time of 20 s. The withdrawal latency was averaged for each animal’s paw on each day.

#### FA test

The intraplantar FA test was performed as described^80^: in brief, FA (5%, 20 μl) was injected into the plantar surface of one hindpaw, and the duration of nocifensive behaviours including lifting, licking or flinching of the injected paw was measured in 5-min bins for a duration of 50 min after injection.

#### Morris water maze

A standard hidden platform protocol was employed. The circular pool (radius = 85 cm) was filled with opaque water to a height of 35 cm and a circular escape platform (radius = 5 cm) was submerged 1 cm below the water surface at a constant position in the centre of the North-West (NW) quadrant during training. The pool openly faced the testing room which provided ample distal cues for visual spatial navigation. To reduce stress effects, mice were habituated to the maze 24 h before training (four trials, visible platform located once in every quadrant). Training consisted of four daily trials on 7 d split into 2 and 5 consecutive days with 2 d of rest in-between. Cages were placed under infrared lamps to prevent hypothermia. Animals were introduced to the pool from start positions East (E), South-East (SE), South (S) and South-West (SW) to avoid close initial proximity to platform. Starting positions were block-randomized with all possible starting positions in random order on every day. After mounting the platform, animals were left there for 15 s. Cut-off time for trials was set to 60 s after which animals failing to locate the platform were guided with a wooden rod and left there for 15 s. One probe trial of 120 s with the platform removed from the pool was conducted 24 h after the last training day. Here, animals entered the pool from position SE.

### Whole-cell patch clamp

Mice were killed by injecting 200 μl of ketamine/xylacine (ketamine: 220 mg kg^−^^1^, Ketavet, Zoetis; and xylazine 16 mg kg^−^^1^, Rompun, Bayer) in PBS and the extracted lumbar spinal cord portion was embedded in 2% low-meting-point agarose (Bio&SELL) and sliced with a vibratome (Leica VT1200S) into 300-µm sections using a slicing solution containing (in mM): sucrose, 191; K-gluconate, 0.75; KH_2_PO_4_, 1.25; NaHCO_3_, 3; d-glucose, 20; myo-inositol, 3; ascorbic acid, 1; choline bicarbonate, 23; ethyl pyruvate, 5; CaCl_2_, 1; MgSO_4_, 4. Slices were allowed to recover for 30 min at 32 °C in recording artificial cerebrospinal fluid (aCSF) containing (in mM): NaCl, 121; KCl, 3; NaH_2_PO_4_, 1.25; NaHCO_3_, 25; d-glucose, 15; ascorbic acid, 1; MgCl_2_, 1.1; myo-inositol, 3; CaCl_2_, 2.2; ethyl pyruvate, 5. Following recovery, slices were placed into the recording chamber and superfused at ~2 ml min^−^^1^ with oxygenated recording aCSF. Whole-cell recordings from random L1 neurons were performed using a patch-clamp amplifier (MultiClamp 700B, Axon Instruments, Molecular Devices) and online data acquisition was performed with pClamp 11 (Axon Instruments). The data were low-pass-filtered at 10 kHz and sampled at a rate of 20 kHz. Electrodes (4–8 MΩ) were pulled from borosilicate glass capillaries (O.D. 1.5 mm, I.D. 0.86 mm, Sutter Instruments). Intrinsic firing properties of L1 neurons were recorded in the presence of 10 mM CNQX, 50 mM AP-V and 5 mM gabazine (Hello Bio), and using an internal solution containing (in mM): K-gluconate, 120; HEPES, 40; MgCl_2_, 5; Na_2_ATP, 2; NaGTP, 0.3. In some experiments aCSF was additionally supplemented with 4-CIN (Sigma), AR-C155858 (Tocris), l-lactate (Sigma) and Sodium Pyruvate (Sigma). In naive animals, recorded neurons were randomly sampled from both dorsal horns; in CFA-injected animals, only the ipsilateral dorsal horn was sampled. Series resistance (*R*_s_) was typically 10–30 MΩ across experiments. In current clamp recordings, pipette capacitance compensation and bridge balance were applied before the recording protocol. Current steps to record rheobase and firing properties were applied for 500 ms from 0 to 120 pA and at 2.5-pA intervals. The liquid junction potential between external and internal solutions of ~12.8 mV was not corrected for.

### Seahorse assay

#### Sample preparation

Mice (3–5 weeks old) were killed by ketamine/xylacine injection and the extracted lumbar spinal cord portion was embedded in 2% low-meting-point agarose (Bio&SELL) and sliced with a vibratome (Leica VT1200S) into 220-µm sections using a slicing solution continuously oxygenated, containing (in mM): sucrose, 191; K-gluconate, 0.75; KH_2_PO_4_, 1.25; NaHCO_3_, 3; d-glucose, 20; myo-inositol, 3; ascorbic acid, 1; choline bicarbonate, 23; ethyl pyruvate, 5; CaCl_2_, 1; MgSO_4_, 4. Seahorse assay in spinal cord tissue was adapted from a previously described protocol^41^ with some modifications. Briefly, slices were transferred to a holding chamber containing continuously oxygenated aCSF (120 mM NaCl, 3.5 mM KCl, 1.3 mM CaCl_2_, 1 mM MgCl_2_, 0.4 mM KH_2_PO_4_, 5 mM HEPES and 10 mM d-glucose; pH 7.4) and allowed to recover for 30 min at room temperature. Sections were individually transferred to a biopsy chamber containing fresh oxygenated aCSF. A rapid-core biopsy and sampling punch with plunger system (500 µm; Micro-to-Nano) was used to take a sample of the dorsal horn. For naive animals, samples were taken from both dorsal horns. In CFA-injected animals, only the ipsilateral dorsal horn was sampled. Punches were ejected directly into an XFe96 Cell Culture Microplate (Agilent Seahorse XF, XF96 FluxPack) based on a predetermined plate layout. Each well contained 180 µl of room temperature assay media (aCSF supplemented with 0.6 mM pyruvate and 4 mg ml^−^^1^ BSA). After loading all biopsy samples, each well was visually inspected to ensure that the punches were submerged and centred at the bottom of the well. The XFe96 Cell Culture Microplate was then incubated at 37 °C in a non-CO_2_ incubator for approximately 30 min. During this incubation period, 10× concentrations of assay drugs (prepared in aCSF) were loaded into their respective injection ports of an XFe96 Extracellular Flux Assay sensor cartridge (previously hydrated for 24 h with Agilent Seahorse XF Calibrant solution at 37 °C in a non-CO_2_ incubator). The sensor cartridge containing the study drugs was then inserted into the analyser for calibration. Once the analyser was calibrated, the calibration plate was replaced by the microplate containing the tissue punches and the assay protocol was initiated.

#### Mitochondrial respiration and glycolysis assay

Mitochondrial respiration and glycolysis assay were measured using the Seahorse Bioanalyzer (Agilent Seahorse, XF96 Bioanalyzer)^40^.

For the mitochondrial respiration, oligomycin (10 µM final), FCCP (15 μM final) and a combination of rotenone and antimycin-A (20 mM and 10 mM final, respectively) were used. The bioanalyzer was calibrated and the assay was performed using the Mito Stress Test protocol as suggested by the manufacturer (Agilent Seahorse Bioscience). The assay was run on one plate with 5–10 replicates per condition.

For the glycolysis assay, the same aCSF media as before was used, with the exception that it did not contain glucose and it was supplemented with 2 mM glutamine and 1 mM sodium pyruvate. Injections of glucose with KCl (10 mM final and 25 mM final, respectively), oligomycin (10 μM final) and 2-deoxy-d-glucose (50 mM final) were diluted in the Agilent Seahorse XF Assay Medium and loaded onto ports A, B and C, respectively. The bioanalyzer was calibrated and the assay was performed using the Glycolytic Stress Test protocol as suggested by the manufacturer (Agilent Seahorse Bioscience). The assay was run on one plate with 5–10 replicates per condition

Seahorse Wave software was used to analyse metabolic data generated from both assays. The data from each assay were normalized to the total protein content with a Pierce BCA Protein Assay Kit following the manufacturer’s instructions.

### Calcium imaging

The coding sequence for jRGECO1a (refs. ^81,82^), a kind gift from Rolf Sprengel, was subcloned using standard procedures into an expression plasmid containing a CaMKIIa promoter and targeted to the nucleus via a tripartite NLS with the addition of a Flag tag^83^, resulting in the pAAV-CKIIa-jRGECO1a.NLS.Flag construct. Serotype 2/1 recombinant adeno-associated viral particles of the construct pAAV-CKIIa-jRGECO1a.NLS were packaged and purified as described^84^ with additional purification of viral particles from the media via polyethylene glycol 8000 precipitation. Briefly, HEK293 cells (Stratagene, 240073; RRID:DVDL_6871) were grown in high-glucose-containing DMEM (Gibco, 41965062) supplemented with 10% heat-inactivated FBS (Gibco, 10270106), sodium pyruvate (Sigma Aldrich, S8636), non-essential amino acids (Gibco, 11140050) and antibiotics. Before transfection by standard calcium phosphate precipitation, the medium was replaced with IMDM (Gibco, 21980065) containing 5% FBS and no antibiotics. After transfection, cells were returned to DMEM. Cells were collected by low-speed centrifugation and resuspended in 150 mM NaCl/20 mM Tris-HCl. Viral particles in the medium were precipitated with the addition of a one-quarter volume of 40% polyethylene glycol 8000 (Merck, P5413) in 400 mM NaCl, pH 7.4, collected by centrifugation and resuspended in 150 mM NaCl/20 mM Tris-HCl. Cells were lysed by 0.5% sodium deoxycholate in the presence of nuclease (Thermo Fisher Scientific, 88701). Recombinant AAVs (rAAV) were subsequently purified on heparin affinity columns (hiTrap Heparin HP; VWR, 17-0406-01) and concentrated via Amicon Ultra-4 centrifugal filter devices (Millipore, UFC8100).

rAAV particles mediating the expression in excitatory neurons of nuclearly localized jRGECO1a, a red fluorescent genetically encoded calcium indicator (CKIIa-jRGECO1a.NLS.Flag)^81^^,82^, were injected bilaterally into the lumbar spinal cord dorsal horn of 5-week-old C57Bl/6N mice (Charles River Laboratories). Mice were anaesthetised with a mixture of fentanyl (50 μg kg^−^^1^), midazolam (5 mg kg^−^^1^) and medetomidine (0.5 mg kg^−^^1^), and the tissue between thoracic vertebrae T12 and T13 and thoracic and lumbar vertebrae T13 and L1—corresponding to spinal lumbar segments L3/L4 and L5/L6, respectively—dissected to reveal the spinal cord. The dura mater was opened to improve needle penetration, and a 36-gauge bevelled ‘NanoFil’ needle mounted in a 10-μl Hamilton syringe (World Precision Instruments) was slowly advanced into the dorsal spinal parenchyma and then left in place for 5 min before viral infusion. Then, 500 nl of rAAV stock corresponding to 0.5 × 10^9^ (CKIIa-jRGECO1a.NLS.Flag) viral particles was subsequently injected at a flow rate of 100 nl min^−^^1^ using a microprocessor-controlled minipump. The needle was left in place for 5 min after the injection was complete to allow for viral penetration into the tissue. Each mouse received a total of four viral infusions. The wounds above the spinal cord were subsequently covered with gelatin foam (Gelita-Spon Standard, Gelita Medical, GS-110) and the skin closed with sutures. Anaesthesia was reversed using a mixture of atipamezole (0.75 mg kg^−^^1^), flumazenil (0.5 mg kg^−^^1^) and burprenorphine (0.1 mg kg^−^^1^). Mice additionally received 5.0 mg kg^−^^1^ carprofen analgesic before the initiation of surgical procedures. After the procedure, mice were placed overnight on a heating plate set to 39 °C and provided with wet food pellets.

At 3–4 weeks following viral infusions, acute spinal cord slices from spinal cord segments L3–L5 of rAAV-injected mice were prepared as described for the Seahorse analyses and imaged. At least 30 min before imaging, slices were placed in continuously carbogenated room temperature starvation aCSF containing (in mM): 120 NaCl, 3.5 KCl, 0.4 KH_2_PO_4_, 1 MgCl_2_, 5 HEPES, 1.3 CaCl_2_, 2 l-glutamine and 1 sodium pyruvate. For imaging, slices were transferred to a recording chamber perfused with continuously carbogenated starvation aCSF and secured with a platinum ring with nylon strings. During imaging, starvation aCSF was exchanged for continuously carbogenated high-potassium stimulation aCSF containing (in mM): 120 NaCl, 25 KCl, 0.4 KH_2_PO_4_, 10 d-glucose, 1 MgCl_2_, 5 HEPES, 1.3 CaCl_2_, 2 l-glutamine and 1 sodium pyruvate. JRGECO1a.NLS was excited using a 550-nm CoolLED light source (545 ± 15 nm), and fluorescence emission (>570 nm) acquired at a rate of 1 Hz with a back-illuminated frame-transfer EM-CCD camera (ImageEM X2; Hamamatsu) through a ×20 water-immersion objective (XLM PlanFluor 0.95W, Olympus) on an upright microscope (Olympus BX51WI) connected to a software interface (Visiview; Visitron Systems). Image sequences were imported into ImageJ (Fiji RRID:SCR_002285) and, when necessary, processed with the ‘Template Matching’ plugin to correct for slice movement during the recording. Regions of interest were manually drawn around larger regions encompassing nearly the entire field of view within laminae I/II/III containing fluorescently labelled cells and processes, and fluorescence intensity changes measured over time. Further data analysis was carried out using Igor Pro (WaveMetrics, RRID:SCR_000325). Photobleaching of the jRGECO1a signal was corrected for using a simple exponential fit to the baseline signal, and bleaching-corrected jRGECO1a fluorescence intensity expressed as the percentage change with respect to baseline in the 10–15 s before stimulation with high-potassium aCSF (% Δ*F*/*F*) and quantified using the peak amplitude above baseline in the first 2 or 4 min after the beginning of the stimulation.

### Statistical analysis

Sample size selection: no statistical methods were used to predetermine sample sizes but our sample sizes are similar to those reported in previous publications for biochemical and metabolic analysis (PMID: 35140588; PMID: 28515312), behavioural assays (PMID: 26291162, PMID: 27306409), Seahorse assays (PMID: 32764697) and electrophysiological experiments (PMID: 26291162; PMID: 27853254). Vivarium-housed mice with the respective/indicated genotypes were randomly allocated into sex-balanced experimental groups.

#### Data analysis

The sequencing reads were aligned by EMBL Genecore to mouse genome Mm10. FastQC was used for quality check and the differential expression analysis was performed with R following the Bioconductor RNA-seq workflow developed by Love et al.^85^. The following packages were used during the data analysis: DESeq2, Rsamtools, GenomicFeatures, TxDb.Mmusculus.UCSC.mm10.ensGene, GenomicAlignments, AnnotationDbi, org.Mm.eg.db. Data were analysed using R version for Linux 3.6.1 (https://www.r-project.org), RStudio for Linux v.1.1.463, RStudio for Windows v.4.1.2 and Matlab for Windows v.R2016a-2020a (MathWorks). Statistical tests were performed using GraphPad Prism for Windows v.7.00-8.0.1 or RStudio for Windows v.4.1.2. Results are presented as mean ± s.e.m. unless indicated otherwise. Distribution of data was assayed using the Kolmogorow–Smirnow normality test, the D’Agostino and Pearson omnibus normality test and the Shapiro–Wilk normality test. For statistical testing of data with only two groups, two-tailed Student’s *t*-test was used. In the case that there were more than two groups with only one source of variation, a one-way analysis of variance (ANOVA) followed by Tukey’s multiple comparison test was used. When comparing two or more groups with more than one source of variation, a two-way ANOVA followed by Bonferroni or Sidak’s post hoc test was used. *P < 0.05, **P < 0.01, ***P < 0.005, ****P < 0.001.

### Data exclusion

In electrophysiological experiments, cells that displayed a considerable variation in membrane resistance or series resistance during the experiment (>50% and >20%, respectively) were excluded from the analysis. For Seahorse analysis, samples in which average baseline OCR was below 20 pg min^−^^1^ after 5 min of recording were excluded. Also, for mitochondrial parameters, samples that did not react to oligomycin, that is, OCR decreased less than 50%, were also excluded. In all experiments, the animal genotype was tested before allocating them into groups and, in some instances, again verified after concluding the experiment; when a genotype was mistaken, the animal was excluded from the experiment or (when possible) allocated, retrospectively, to the correct group.

### Reporting summary

Further information on research design is available in the Nature Portfolio Reporting Summary linked to this article.

### Supplementary information

Reporting Summary

## Data Availability

Individual data points are represented throughout all figures. Data distribution was assumed to be normal but this was not formally tested. The associated data are provided as source data files, with all data that are presented in each main and Extended Data figure included in subfolders named correspondingly in the source data folder that can be found on the HeiData server of Heidelberg University (https://heidata.uni-heidelberg.de/) at 10.11588/data/AMDH7G. The RNA-seq dataset generated as part of Fig. 1 and Extended Data Fig. 1 can be accessed at https://www.ebi.ac.uk/ with the following ArrayExpress accession number: E-MTAB-13734. Statistical analysis and reproducibility: experiments showing representative micrographs (Fig. 1c,d and Extended Data Figs. 1d–f,i, 3b and 4g) have been performed at least twice independently if not stated otherwise.

## Code availability

We used a custom written code in R for analysis of electrophysiology results shown in Fig. 6 and Extended Data Fig. 6. This code is freely available at GitHub at the following link: https://github.com/wambroziak/Neuron_astrocyte_metabolic_coupling_ephys. RNA-seq analysis code is provided in the corresponding source data files.
