# Supplementary material for: Neuron–astrocyte metabolic coupling facilitates spinal plasticity and maintenance of inflammatory pain
Source: Nat Metab. 2024 Mar 5;6(3):494–513. doi: 10.1038/s42255-024-01001-2 (PMC10963271; doi:10.1038/s42255-024-01001-2)
Supplement: Supplementary file 1 — Reporting Summary [file 42255_2024_1001_MOESM1_ESM.pdf]

nature portfolio

Corresponding author(s): Jan Siemens

Last updated by author(s): Jan 15, 2024

Reporting Summary

Nature Portfolio wishes to improve the reproducibility of the work that we publish. This form provides structure for consistency and transparency in reporting. For further information on Nature Portfolio policies, see our Editorial Policies and the Editorial Policy Checklist.

Statistics

For all statistical analyses, confirm that the following items are present in the figure legend, table legend, main text, or Methods section.

n/a	Confirmed	
☐	☒	The exact sample size (*n*) for each experimental group/condition, given as a discrete number and unit of measurement
☐	☒	A statement on whether measurements were taken from distinct samples or whether the same sample was measured repeatedly
☐	☒	The statistical test(s) used AND whether they are one- or two-sided<br>*Only common tests should be described solely by name; describe more complex techniques in the Methods section.*
☒	☐	A description of all covariates tested
☐	☒	A description of any assumptions or corrections, such as tests of normality and adjustment for multiple comparisons
☐	☒	A full description of the statistical parameters including central tendency (e.g. means) or other basic estimates (e.g. regression coefficient) AND variation (e.g. standard deviation) or associated estimates of uncertainty (e.g. confidence intervals)
☐	☒	For null hypothesis testing, the test statistic (e.g. *F*, *t*, *r*) with confidence intervals, effect sizes, degrees of freedom and *P* value noted<br>*Give P values as exact values whenever suitable.*
☒	☐	For Bayesian analysis, information on the choice of priors and Markov chain Monte Carlo settings
☒	☐	For hierarchical and complex designs, identification of the appropriate level for tests and full reporting of outcomes
☒	☐	Estimates of effect sizes (e.g. Cohen's *d*, Pearson's *r*), indicating how they were calculated

Our web collection on statistics for biologists contains articles on many of the points above.

Software and code

Policy information about availability of computer code

Data collection	RNA Sequencing was performed using MiSeq, Illumina and FastQC was used for quality check of sequencing reads. Glycogen, Lactate, Enzymatic Assays and protein data collection was performed with i-control v1.12, Tecan Austria GmbH. qPCR data was collected using LightCycler® 96 v1.1.0.1320, Roche. Confocal microscopy data was collected using NIS-Element AR, Nikon. Seahorse data was collected using XF software v1.4.2.3, Agilent Technologies. Electrophysiological data was collected using pClamp v11.0.0.03, Molecular Devices. Calcium Imaging data was collected with Visiview, Visitron Systems.
Data analysis	The differential expression Analysis was performed with R following the Bioconductor RNA-Seq workflow developed by Love et al., 2015 using R version for Linux v3.6.1., RStudio for Linux v1.1.463 and Matlab for Windows vR2016a-2020a, MathWorks. Electrophysiological data was analysed with R Studio for Windows v4.1.2 and GraphPad Prism for Windows v7.00-8.0.1, GraphPad software. Seahorse analysis was performed with Wave v2.6.3.5, Agilent Technologies. Microscopy data analysis was performed using Fiji v2.9.0, ImageJ. Ptg quantification was performed with Ilastik v1.4.0. Calcium Imaging data was analysed with Igor Pro, WaveMetrics. All other quantitative data analysis was performed with GraphPad Prism for Windows v7.00-8.0.1, GraphPad software.

For manuscripts utilizing custom algorithms or software that are central to the research but not yet described in published literature, software must be made available to editors and reviewers. We strongly encourage code deposition in a community repository (e.g. GitHub). See the Nature Portfolio guidelines for submitting code & software for further information.

Data

Policy information about availability of data

All manuscripts must include a data availability statement. This statement should provide the following information, where applicable:

- Accession codes, unique identifiers, or web links for publicly available datasets
- A description of any restrictions on data availability
- For clinical datasets or third party data, please ensure that the statement adheres to our policy

Associated data is provided as Source Data Files with each main or Extended Data figure which is available from the source data supplementary files and publicly available from HeiData:
RNA-Seq dataset generated in this study can be accessed from https://www.ebi.ac.uk/ with the following ArrayExpress accession number: E-MTAB-13734.

Research involving human participants, their data, or biological material

Policy information about studies with human participants or human data. See also policy information about sex, gender (identity/presentation), and sexual orientation and race, ethnicity and racism.

Reporting on sex and gender	N/A
Reporting on race, ethnicity, or other socially relevant groupings	N/A
Population characteristics	N/A
Recruitment	N/A
Ethics oversight	N/A

Note that full information on the approval of the study protocol must also be provided in the manuscript.

Field-specific reporting

Please select the one below that is the best fit for your research. If you are not sure, read the appropriate sections before making your selection.

☒ Life sciences ☐ Behavioural & social sciences ☐ Ecological, evolutionary & environmental sciences

For a reference copy of the document with all sections, see nature.com/documents/nr-reporting-summary-flat.pdf

Life sciences study design

All studies must disclose on these points even when the disclosure is negative.

Sample size	For electrophysiology experiments where the frequency of action potentials was measured, the minimum sample size based on the effect size calculated was 5 cells. For other electrophysiological recordings as well as in vivo behavioural experiments, molecular, biochemical and metabolical analysis as well as for Seahorse Assays, sample size was determined based on comparable literature data.
Data exclusions	In electrophysiological experiments, cell recordings where membrane resistance or series resistance changed considerably during experiment (>50% and >20% respectively) were excluded from analysis. For Seahorse analysis, samples in which average baseline OCR was below 20 pg/min after 5 minutes of recording was excluded. Also, for mitochondrial parameters, samples that did not react to Oligomycin, i.e OCR decreased less than 50% were excluded as well. In all experiments, the animals genotype was tested before allocating them into groups and after concluding the experiment; when genotype was mistaken, the animals was excluded from the experiment or (when possible) allocated retrospectively to the correct group.
Replication	Electrophysiological experiments were usually done with cell recordings from at least three mice. In vivo behavioural experiments were conducted according to comparable literature data (minimum of 5 animals per group). At least two rounds of testing were conducted per type of experiment to exclude that something was wrong in the parameters used and the results were therefore replicable. Number of animals tested per each experiment is indicated in the figure legends (as N=XX).
Randomization	In experiments where genotype was not a variable, similar age mice were randomly assigned to each group. In experiments where genotype was a variable, mice were assigned to each group based on their genotype but randomly sampled from core colonies. Only in behavioral studies litter mates were actively selected.
Blinding	On all behavioural experiments investigators were blinded to group allocation during data collection and until the end of data analysis. For all other experiments, investigators where blinded to group allocation until sample collection, after which samples were processed and analysed indistinctively of group until group allocation at the end of data analysis. In both cases, data was collected indistinctively of grouping.

Reporting for specific materials, systems and methods

We require information from authors about some types of materials, experimental systems and methods used in many studies. Here, indicate whether each material, system or method listed is relevant to your study. If you are not sure if a list item applies to your research, read the appropriate section before selecting a response.

Materials & experimental systems

n/a	Involved in the study
☐	☒ Antibodies
☐	☒ Eukaryotic cell lines
☒	☐ Palaeontology and archaeology
☐	☒ Animals and other organisms
☒	☐ Clinical data
☒	☐ Dual use research of concern
☒	☐ Plants

Methods

n/a	Involved in the study
☒	☐ ChIP-seq
☒	☐ Flow cytometry
☒	☐ MRI-based neuroimaging

Antibodies

Antibodies used	1-pS6 #2215 (Cell Signaling); 1:1000<br>2-GFAP #3670 (Cell Signaling) 1:500<br>3-NeuN #D4G40 (Cell Signaling) 1:2000<br>4-IBA1 #019-19741 (Wako) 1:500<br>5-GFAP #173004 (Synaptic Systems) 1:1000<br>6-GFP #600-101-215 (Rockland) 1:1000<br>7-mCherry #AB0040-500 (Sicgen) 1:5000<br>8-Sox9 #ab185966 (Abcam) 1:1500<br>9-Donkey α-Rabbit, Alexa Fluor 488, #A21206 (Invitrogen) 1:1000<br>10-Goat α-mouse Alexa Fluor 488, #A11001 (Invitrogen) 1:1000<br>11-Donkey α-Goat Alexa Fluor 488 #A11055 (Invitrogen) 1:1000<br>12-Donkey α-Goat Alexa Fluor 555 #A21432 (Invitrogen) 1:1000<br>13-Donkey α-Mouse Alexa Fluor 594 #711585150 (Dianova) 1:1000
Validation	1-pS6 #2215 (Cell Signaling) - Validated by manufacturer for Western blot with analysis of extracts from 293 cells, untreated or treated with 20% FBS.<br>2-GFAP #3670 (Cell Signaling) - Validated by manufacturer for Immunofluorescence (IF) with analysis of Confocal IF image of rat hippocampus.<br>3-NeuN #D4G40 (Cell Signaling) - Validated by manufacturer for IF with analysis of Confocal IF image of mouse hippocampus, cortex, and cerebellum.<br>4-IBA1 #019-19741 (Wako) - Validated by manufacturer for IF with analysis of IF image of mouse cerebellum, Nucleus accumbens core and spinal cord.<br>5-GFAP #173004 (Synaptic Systems) - Validated by manufacturer for IF with analysis of IF image of mouse hippocampus.<br>6-GFP #600-101-215 (Rockland) - Validated by manufacturer for IF with analysis of IF image of GFP-positive transgenic mouse brain and in-house with IF analysis of GFP-positive versus negative mice.<br>7-mCherry #AB0040-500 (Sicgen) - Validated in house with IF analysis of mCherry-positive vs negative mice.<br>8-Sox9 #ab185966 (Abcam) - Validated by manufacturer for Western blot with analysis of extracts of SW480 cells versus HeLa cells, and for IF through IF analysis of F9 (Mouse embryonic testicular cancer cell line) cells.

Eukaryotic cell lines

Policy information about cell lines and Sex and Gender in Research

Cell line source(s)	HEK AAV-293 cells that were used to generate serotype 1/2 rAAV particles were obtained from Stratagene (#240073).
Authentication	HEK AAV-293 cells used in this study were authenticated by Statagene.
Mycoplasma contamination	Cell lines were not tested for mycoplasma contamination.
Commonly misidentified lines<br>(See ICLAC register)	No commonly misidentified cell lines were used in this study

Animals and other research organisms

Policy information about studies involving animals; ARRIVE guidelines recommended for reporting animal research, and Sex and Gender in Research

Laboratory animals	Mice (mus musculus) of both sexes were used for experiments. Lines used: Aldh1L1-(Cre/ERT2): B6N.FVB-Tg(Aldh1l1-Cre/

Laboratory animals	ERT2)-1Khakh/J; cPTG(-/-): B6N.FVB-Tg(Aldh1l1-Cre/ERT2)-1Khakh/J-PPP1R3C-LoxP; gPTG: PPP1R3C(-/-). For Seahorse Assay, 4-6 week old mice were used. All other experiments performed at a starting age of 8-12 weeks.
Wild animals	No wild animals were used.
Reporting on sex	Sex-based analysis was not performed, based in previous literature suggesting no sex-difference effect on Astrocytic metabolism, but a balanced male/female ratio was used by randomly sampling core colonies. This information has not been collected for this study.
Field-collected samples	No field-collected samples were used
Ethics oversight	All experiments involving animals were approved by the local authorities (Regierungspräsidium Karlsruhe), under animal protocol numbers G-168/15, G-201/16, G-295/21, G-173/21.

Note that full information on the approval of the study protocol must also be provided in the manuscript.

Plants

Seed stocks	N/A
Novel plant genotypes	N/A
Authentication	N/A
